# Spiroindolines Identify the Vesicular Acetylcholine Transporter as a Novel Target for Insecticide Action

**DOI:** 10.1371/journal.pone.0034712

**Published:** 2012-05-01

**Authors:** Ann Sluder, Sheetal Shah, Jérôme Cassayre, Ralph Clover, Peter Maienfisch, Louis-Pierre Molleyres, Elizabeth A. Hirst, Anthony J. Flemming, Min Shi, Penny Cutler, Carole Stanger, Richard S. Roberts, David J. Hughes, Thomas Flury, Michael P. Robinson, Elke Hillesheim, Thomas Pitterna, Fredrik Cederbaum, Paul A. Worthington, Andrew J. Crossthwaite, John D. Windass, Richard A. Currie, Fergus G. P. Earley

**Affiliations:** 1 Cambria Biosciences, Woburn, Massachusetts, United States of America; 2 Syngenta Crop Protection Research, Bracknell, Berkshire, United Kingdom; 3 Syngenta Crop Protection AG, Stein, Switzerland; U. Kentucky, United States of America

## Abstract

The efficacy of all major insecticide classes continues to be eroded by the development of resistance mediated, in part, by selection of alleles encoding insecticide insensitive target proteins. The discovery of new insecticide classes acting at novel protein binding sites is therefore important for the continued protection of the food supply from insect predators, and of human and animal health from insect borne disease. Here we describe a novel class of insecticides (Spiroindolines) encompassing molecules that combine excellent activity against major agricultural pest species with low mammalian toxicity. We confidently assign the vesicular acetylcholine transporter as the molecular target of Spiroindolines through the combination of molecular genetics in model organisms with a pharmacological approach in insect tissues. The vesicular acetylcholine transporter can now be added to the list of validated insecticide targets in the acetylcholine signalling pathway and we anticipate that this will lead to the discovery of novel molecules useful in sustaining agriculture. In addition to their potential as insecticides and nematocides, Spiroindolines represent the only other class of chemical ligands for the vesicular acetylcholine transporter since those based on the discovery of vesamicol over 40 years ago, and as such, have potential to provide more selective tools for PET imaging in the diagnosis of neurodegenerative disease. They also provide novel biochemical tools for studies of the function of this protein family.

## Introduction

Increasing demand for food, fuel and fibre crops from limited agricultural land area will drive more intensive agricultural practices that are more vulnerable to losses from pests and disease. The need for effective control of insect pests in agriculture is therefore becoming more important [1], [2], [3]. Reliable insect control requires the development of novel insecticides that overcome resistance against existing classes in pest populations, and this is not only an issue for all major agrochemical classes, but also has become a critical issue for human and animal health [4], [5], [6]. One form of resistance is a consequence of selection for less sensitive forms of the insecticide target protein, and so insecticides that target novel proteins are valuable developments. Here we report the discovery of an insecticide class that acts at the vesicular acetylcholine transporter, a novel target for insect control. Identification of this protein as the target delivering the insecticidal effect was driven by a forward genetics approach in model organisms and harnessed the gene function knowledge base in the free living nematode Caenorhabditis elegans.

**Figure 1:**
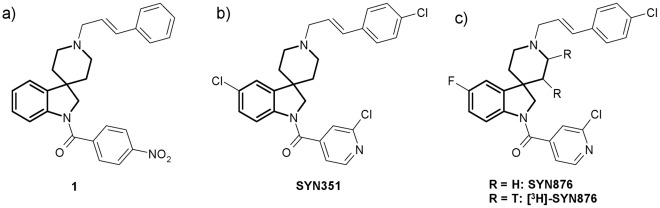
From hit to lead: selected structures of Spiroindolines. a) **1**: insecticidal hit identified through high-throughput screening; b) **SYN351**: lead compound used in studies of resistance in *C.elegans* and *D.melanogaster* (prepared according to path b in Figure 2); c) **SYN876**: lead compound tested in the field for insecticidal activity (prepared according to path a in Figure 2) and used as radioligand ([^3^H]- **SYN876** prepared according to path d in Figure 2).

**Figure 2:**
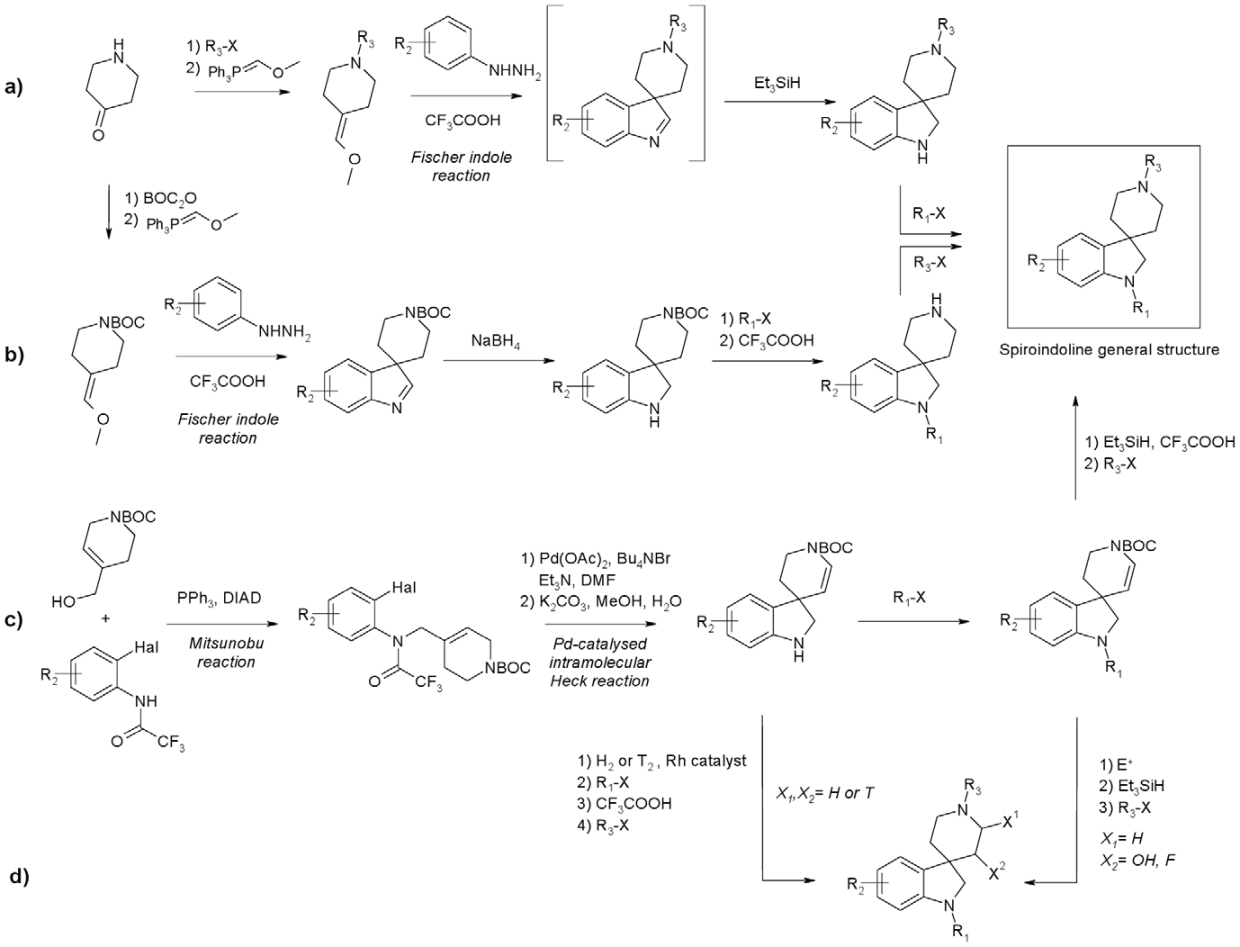
General structure of Spiroindolines and major synthetic routes: a) Fischer Indole synthesis with flexible variation of R_1_; b) alternative Fischer Indole synthesis allowing convergent variation at R_3_; c) alternative, novel synthetic pathway through intramolecular Heck reaction allowing broad variation at R_2_; and d) functionalisation of piperidine ring (X_1_, X_2_ = H, ^3^H, F). Detailed methods are provided in on-line materials.

In nematodes and vertebrates, acetylcholine acts as a fast excitatory neurotransmitter at neuronal synapses and at the neuromuscular junction, whereas in insects its role in this respect is restricted to the central nervous system [7]. Preceding its release into the synapse, acetylcholine is synthesized in the presynaptic terminal and loaded into specialized storage and release vesicles through the action of the vesicular acetylcholine transporter (VAChT), a member of the major facilitator superfamily closely related to the monoamine neurotransmitter transporters and believed to be structurally related to bacterial transporters [8]. In C. elegans, the fruit fly Drosophila melanogaster and mammals, VAChT is encoded by a single gene at a complex locus that also contains the coding sequence for the biosynthetic enzyme cholineacetyltransferase [9].

The acetylcholine signalling pathway has already been successfully exploited by several insecticide classes of major commercial importance acting either as acetylcholinesterase inhibitors (organophosphates and carbamates), which are now in declining use because of resistance and safety issues; or as nicotinic acetylcholine receptor activators (neonicotinoids and spinosyns), for which resistance is an emerging problem for both agriculture and animal health [10], [11].

**Figure 3:**
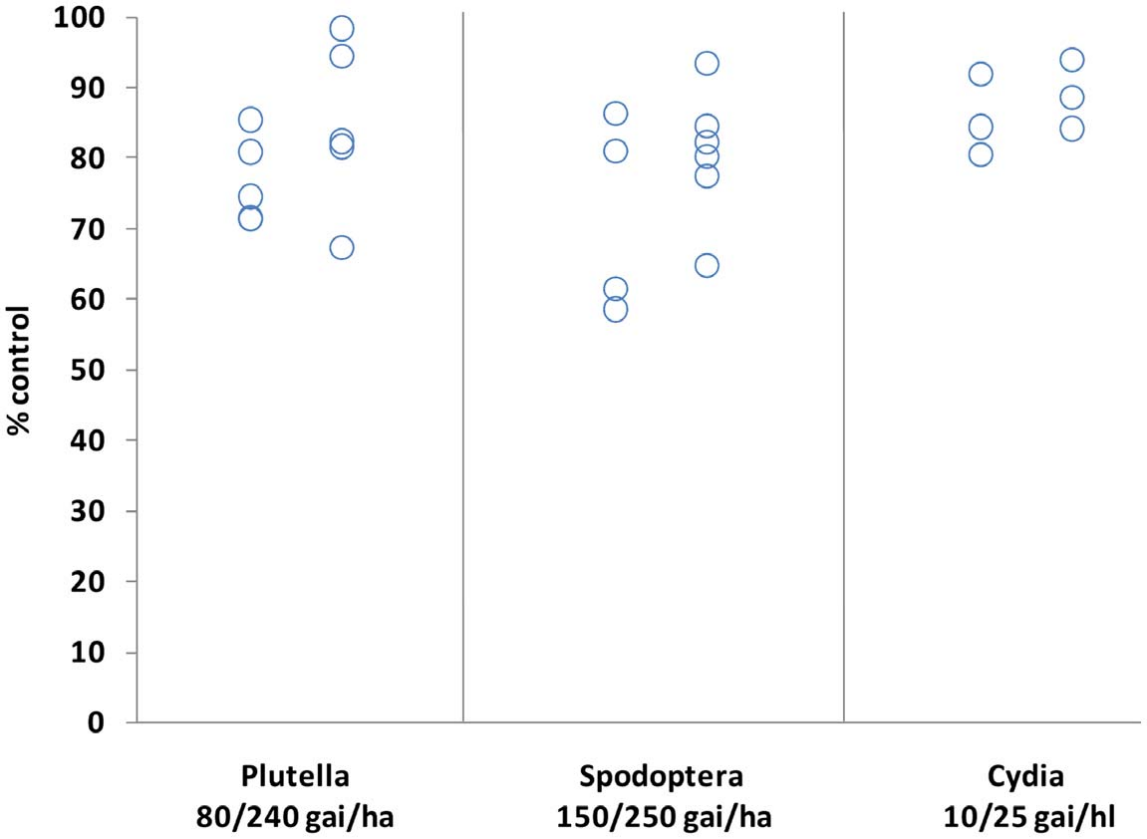
Field efficacy of SYN876 at different application rates, expressed as percent insect control compared with untreated plots. Pest and application rates are given on the x axis. Each point represents data from an independent trial and is an average of multiple assessments. Full details are given in online methods.

## Results

### The Discovery of Insecticidal Spiroindolines and Optimization of their Biological Activity

Organic compounds incorporating the spiro[indoline-3,4'-piperidine] scaffold have been reported to induce several pharmaceutical effects and the scaffold is considered as a “privileged component” of G-protein coupled receptor ligands [12], [13]. High throughput screening of a spiroindoline chemical library for insecticidal activity led to the identification of several insecticidal spiro[indoline-3,4'-piperidine] compounds, including compound **1** (Figure 1), which displayed significant activity against the insect species D. melanogaster, Plutella xylostella (diamond-back moth) and Heliothis virescens (tobacco budworm) at 1000 µg.ml^−1^ (see online Methods and Validation). Intrigued by a possible extension of the privileged nature of this structural scaffold to crop protection research, we embarked on an optimization program around this initial lead which resulted in the identification of highly potent and selective insecticides such as SYN351 and SYN876 [14]. The Fischer-Indole reaction was found to be a reliable route for the synthesis of spiro[indoline-3,4'-piperidine] compounds as previously described [15], [16], and this methodology was improved and applied to the convergent functionalization of the indoline (Figure 2, path a) or the piperidine nitrogen (path b). We also devised a novel route based on an intramolecular Heck reaction for the synthesis of spiroindolines with electron-poor aromatic rings as well as the further functionalization of the piperidine ring (path c) [17]. The latter route was used for the synthesis of the radioligand [3H]-SYN876 after tritiation of the piperidine double bond (path d).

**Figure 4:**
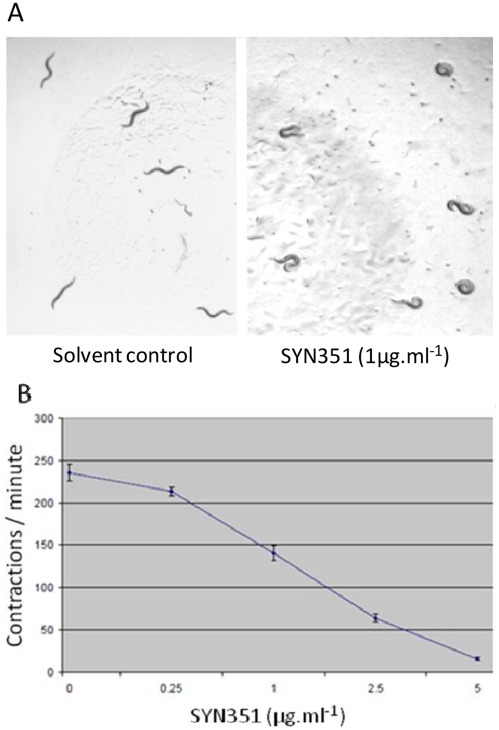
Spiroindolines induce coiling (A) and inhibit pharyngeal pumping (B) in *C. elegans*. Wild-type (N2) animals treated with SYN351 (shown) or SYN876 grow slowly and display a coiling, “loopy” posture during locomotion (right panel) compared to controls (left panel). Pharyngeal pumping rate was determined for four wild-type (N2) animals at each concentration of SYN876, by manually counting the contractions of the posterior bulb of the pharynx over one minute.

The biological activity of a selected set of compounds against Spodoptera littoralis (cotton leafworm), H. virescens and P. xylostella is shown in Table S1. The 4-nitrobenzoyl indoline substituent in compound **1** was replaced by an isonicotinoyl group, giving increased potency and photo stability (Table S1, entry 1). Introduction of a halogen atom on the aromatic ring of the indoline (entry 2) or the cinnamyl cap (entry 3) had a positive and cumulative effect (entry 4) on insecticidal activity that could not be accounted for by changes in activity at the target protein (entries 24 and 26, tables S2 and S3), and so is a probable result of increased metabolic stability [18]. Structure-activity relationships revealed in particular the importance of the cinnamyl substituent at the piperidine nitrogen (Table S1 entries 4, 6 and 7) as well its (E)-double bond geometry (entry 5). With SYN351 and SYN876 (entries 4 and 8), an optimal substitution pattern was found and these two compounds were selected for further biological evaluation and mode of action studies.

**Figure 5:**
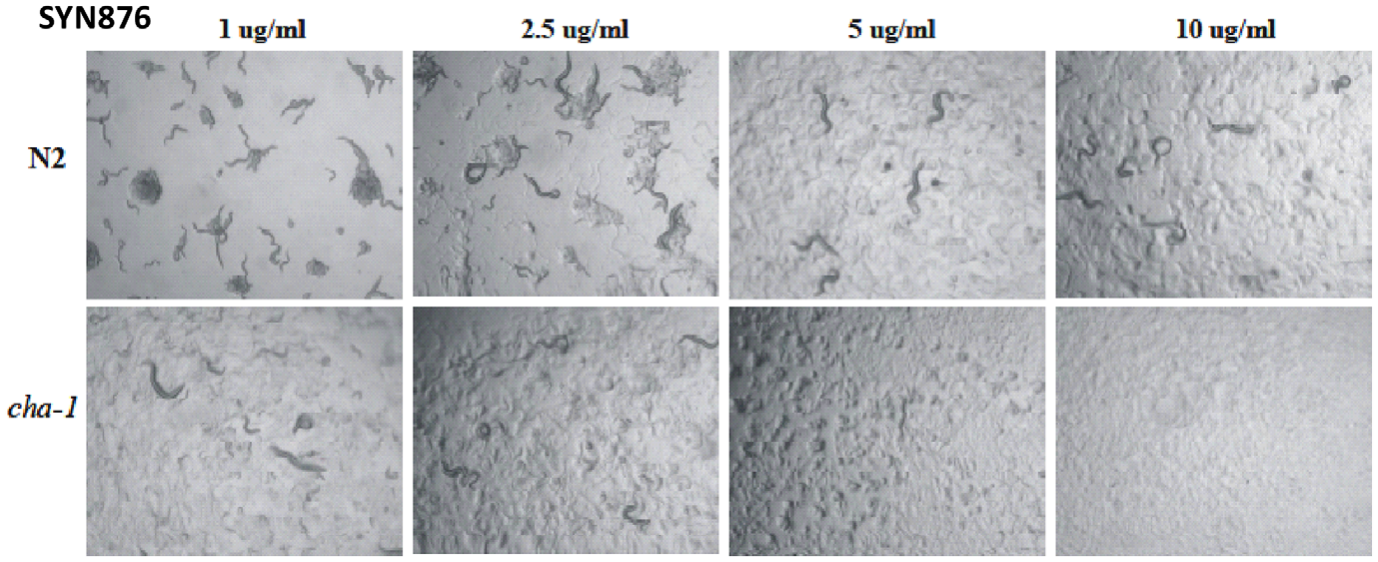
*cha-1(p1152)* is hypersensitive to SYN876. Staged L1 hermaphrodite larvae were plated onto agar culture medium (∼50 animals/well of a 24 well microplate) containing SYN876 added as a concentrated solution in dimethylsulphoxide (DMSO) to give the indicated final concentrations. The final concentration of DMSO in all assays was 1%. Micrographs show growth after 3 days.

**Figure 6:**
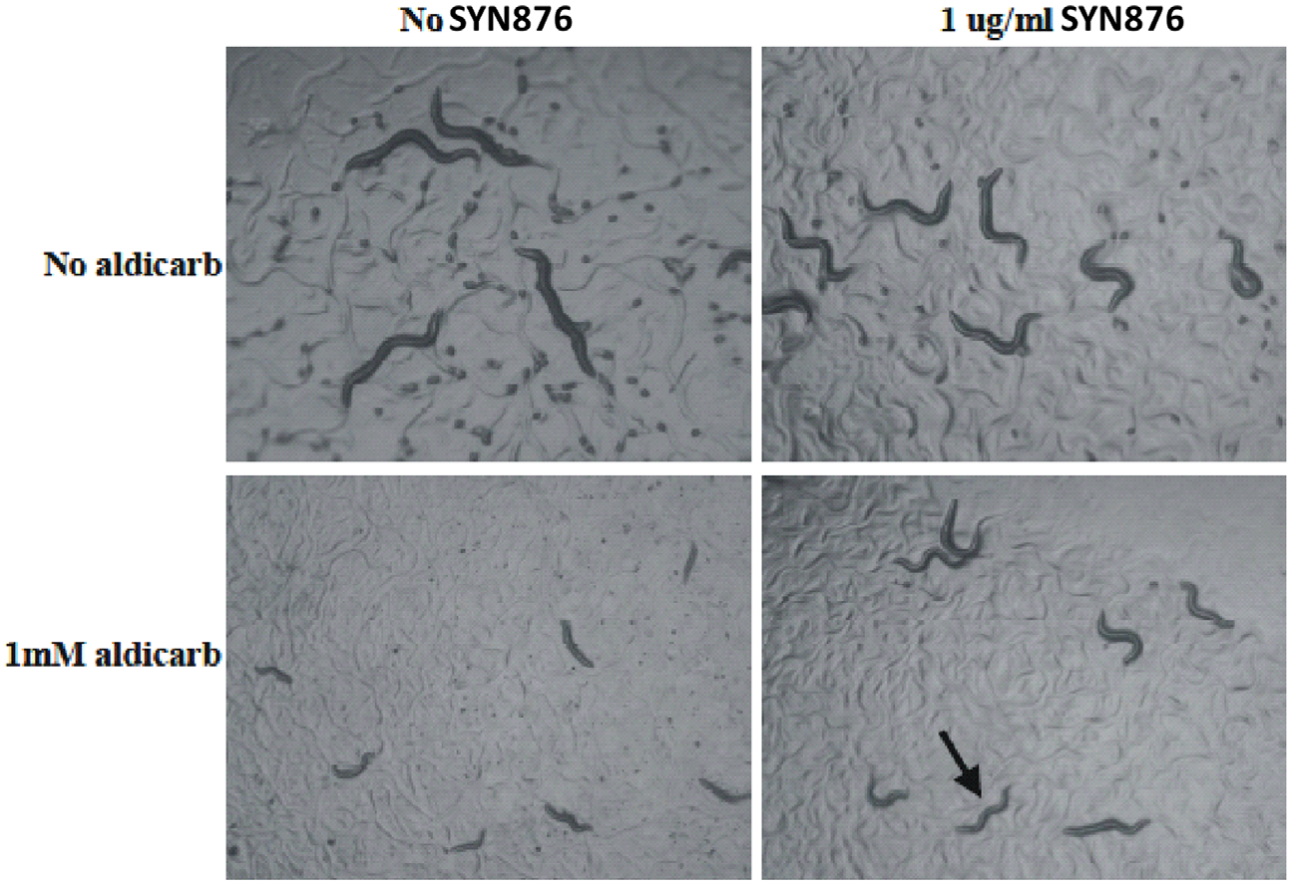
Sublethal doses of SYN876 suppress aldicarb effects. Wild-type *C. elegans* was exposed to the indicated compound concentrations. Worms exposed to 1 mM aldicarb are hypercontracted and exhibit little movement (lower left panel). In the presence of both 1 mM aldicarb and 1 µg.ml^-1^ SYN876 the worms are much less hypercontracted and exhibit fairly normal sinusoidal locomotion (arrow, lower right panel). Worms exposed to 1 mM aldicarb and >2.5 µg/ml SYN876 exhibit movement and growth defects similar to those induced by SYN876 alone (uncoordinated movement and coiling without hypercontraction; not shown).

As a representative member of the family, the lead Spiroindoline SYN876 is slightly basic (pKa 7.88), displays high lipophilicity (logP 5.94), low water solubility (H2O sol. 5 µg.ml^−1^ at pH 6.15), and exhibits moderate photostability (T_50_ 114 mins). Its efficacy in the field against representative lepidopteran pests is shown in Figure 3, and was found to approach or exceed that of commercial standards such as Spinosad [19]. Moreover, besides its high level of insecticidal activity, SYN876 presents a favourable acute oral toxicity in rats (MLD_50_ >200 mg.kg^−1^).

### The Effects of Insecticidal Spiroindolines on Caenorhabditis Elegans and Characterization of Resistant Mutants

Spiroindolines are toxic to C. elegans, in which they induce an uncoordinated “loopy”, coiling locomotion and reduced frequency of pharyngeal pumping (Figure 4), symptoms that are similar to the phenotypes of mutants in which neuromuscular cholinergic signalling is reduced [20]. Animals bearing a partial loss of function mutation in choline acetyltransferase (ChAT), which produce reduced levels of acetylcholine, are hypersensitive to Spiroindolines (Figure 5). One simple explanation for these observations is that the Spiroindolines somehow act to reduce the availability of acetylcholine at the synapse.

To test this hypothesis more directly, we examined the interaction of SYN876 with the cholinesterase inhibitor aldicarb. Exposure to aldicarb leads to muscle hypercontraction and eventual nematode death, and mutants such as cha-1 that produce reduced levels of acetylcholine are resistant to aldicarb since they are more able to tolerate some increase in synaptic acetylcholine levels than wild-type nematodes. Furthermore, low levels of aldicarb suppress the movement and growth defects of these mutants [21]. We therefore reasoned that if the Spiroindolines act to reduce synaptic acetylcholine levels, they would be able to suppress the effects of aldicarb. Consistent with this hypothesis, sub-lethal doses of SYN876 ameliorated the effects of aldicarb exposure (Figure 6).

**Figure 7:**
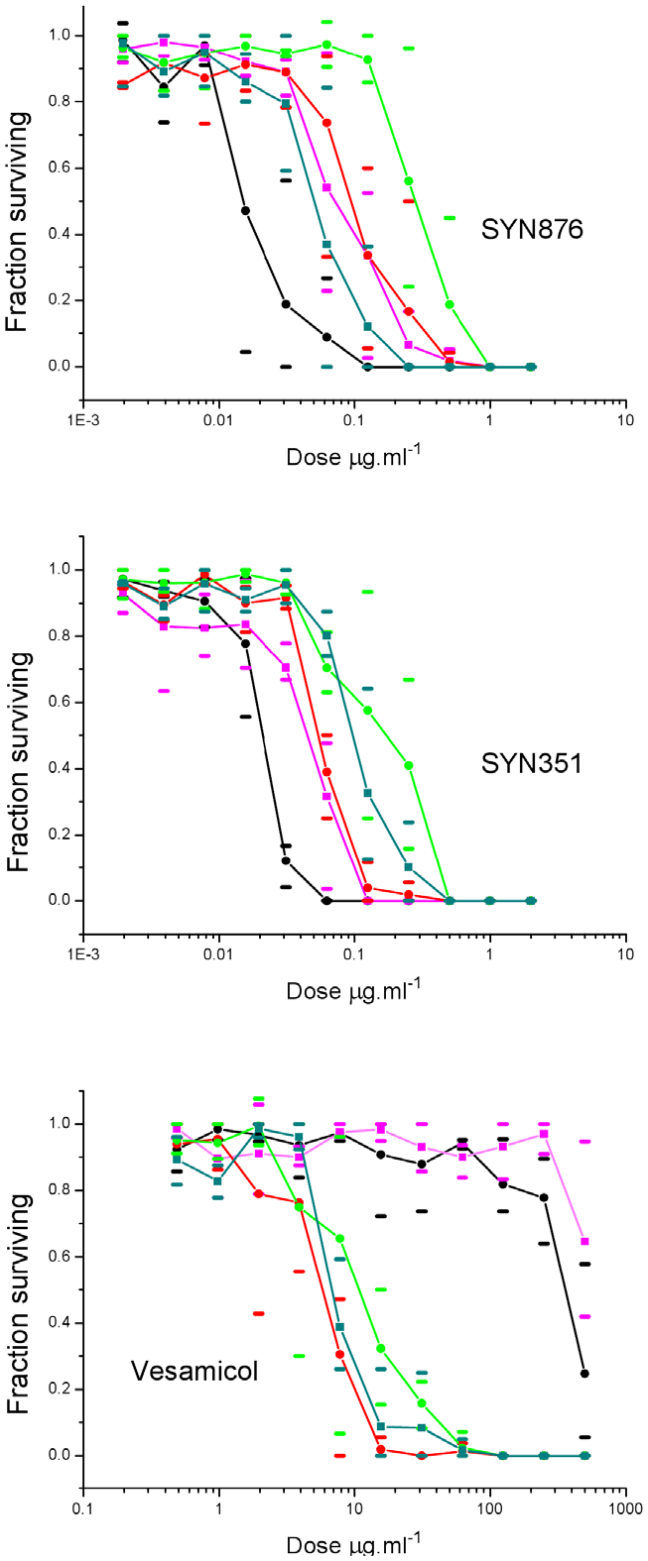
The effect of two Spiroindolines and vesamicol on the survival of parental and mutant *C. elegans* larvae to adulthood. Black lines represent sensitivity of the wild-type strain N2. Coloured lines represent sensitivity of the mutant alleles selected for resistance to SYN351; *cb28* (magenta), *cb29* (red), *cb30* (green), *cb34* (cyan). Solid symbols represent the means, and bars represent the maximum and minimum values for three replicate experiments. The amino acid change for each mutant allele is indicated in Figure 8.

**Figure 8:**
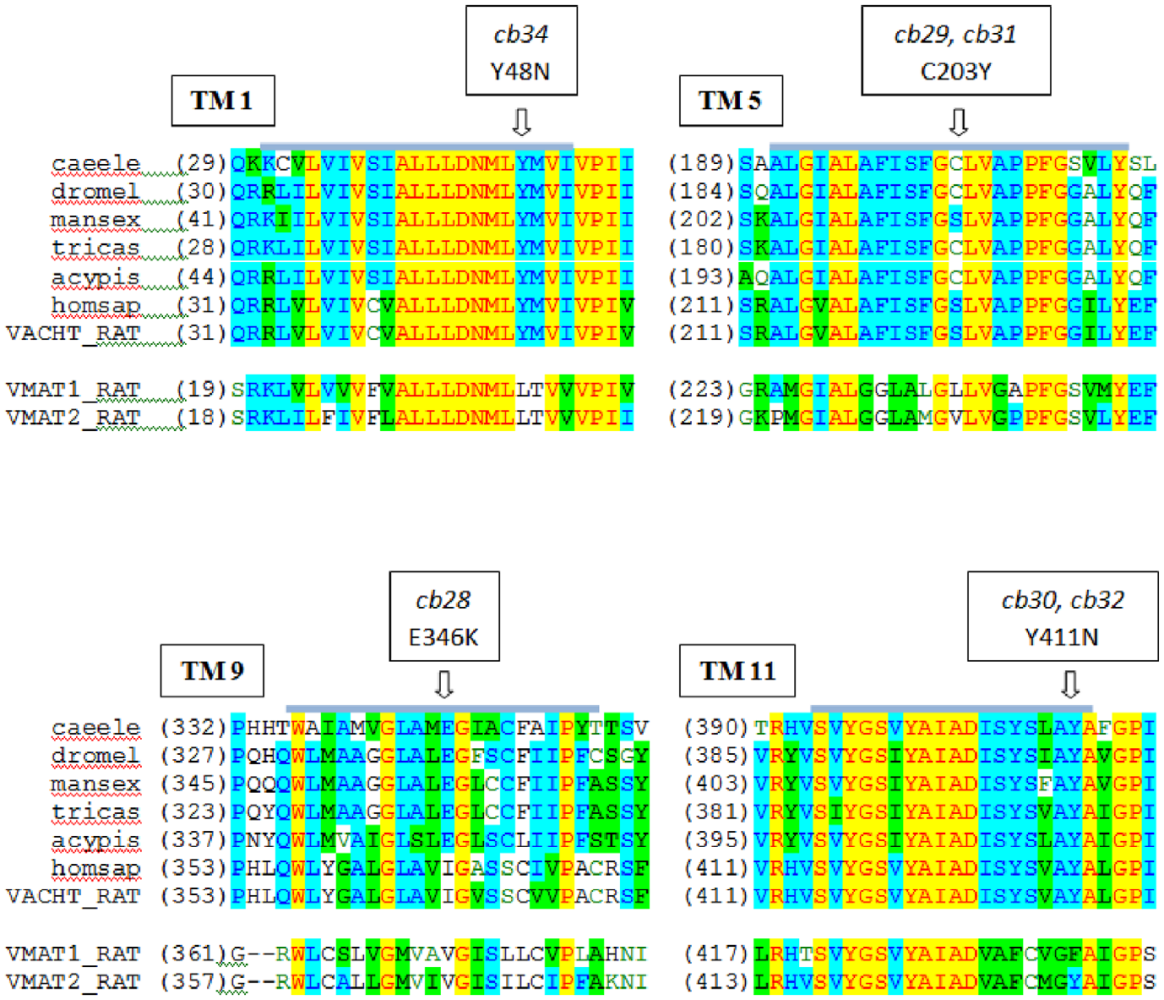
VAChT Protein sequence alignments for transmembrane domains in which amino acid changes gave rise to resistance to SYN351. The *C. elegans* sequence is aligned with those from four insect species; *D. melanogaster* (dromel), *Manduca sexta* (mansex), *Tribolium castanaeum* (tricas) and, *Acyrthosiphon pisum* (acypis); the human (homosap) and rat sequence (VACHT_RAT), and corresponding sequences of the rat monoamine transporters. Predicted transmembrane helices (TM) are marked by grey bars. The sequence changes in *C. elegans unc-17* mutants resistant to Spiroindolines are indicated by the arrows.

Chemical mutagenesis generated C. elegans mutants resistant to Spiroindolines (Figure 7). The low frequency of recovery and genetic dominance of the mutations indicated that resistance was due to gain of function. Mutations conferring resistance mapped to a 5 map unit interval on Chromosome IV that contains the genes for VAChT (unc-17) and ChAT (cha-1) (see Text S1). The apparent requirement for a gain-of-function mutation to confer resistance further suggested that the mutations affected an essential gene (or genes), and it is known that both unc-17 and cha-1 function are required for nematode viability [22], [23]. All mutants characterized had sequence changes in the coding region of unc-17 that resulted in amino acid substitutions (Figure 8), providing compelling evidence that unc-17 is the locus encoding resistance. The C203Y and Y411N mutations were each recovered in two independent lines, suggesting that very few changes allow for both Spiroindoline resistance and nematode viability.

### Generation of Resistance to Spiroindolines in Drosophila Melanogaster

To confirm that the results from nematodes were applicable to insects, we tested whether the same amino acid substitutions could also generate resistance in D. melanogaster. Wild-type, and variant forms of D. melanogaster VAChT (Y49N and E341K in the Drosophila protein corresponding to strong and weak C. elegans resistance alleles cb34 and cb28 respectively) were ectopically expressed using the Gal4-UAS modular misexpression system [24]. Expression of the variants was driven by the cha promoter, thus mimicking the endogenous expression pattern of vacht. Multiple independent lines were tested for each variant and viable insects with no remarkable phenotypes were recovered for all.

Flies over-expressing the wild-type form of the vacht gene, cha>vacht+, were at least 3-fold less susceptible than control genotypes to SYN351-mediated mortality, demonstrating that simple over-expression of VAChT protein conferred resistance to SYN351. Flies over-expressing one mutant form of the vacht gene, cha>vacht^Y49N^, were completely insensitive to all doses of SYN351 tested over an extended period of time (Table 1). These results indicated that a single amino acid change in the VAChT protein is capable of generating very high levels of resistance, and that the expression of VAChT[Y49N] in a wild type background is sufficient to overcome the toxic effect of this compound in Drosophila. Thus the mechanism of resistance to SYN351 translates from worms to flies and relates to the function of VAChT.

None of the flies engineered to over-express the E341K mutant form of the vacht gene, cha>vacht^E341K^, were resistant to SYN351-mediated mortality. These flies did not even exhibit the same level of resistance as Cha>vacht+ flies. One explanation for this observation is that the VAChT E341K variant protein is either not expressed or is unstable relative to the ectopic expression of VAChT+, and this inference was supported by the failure to detect elevated levels of the protein by western blotting [data not shown].

**Table 1:** Resistance to SYN351 generated by ectopic expression of VAChT in *D. melanogaster*.

	Comparisons between test and parental genotypes	Resistance factors (95% confidence limits)
(i)	*Cha>vacht^+(1-3)^: Cha*	3.54 (2.03 – 6.52)
	*Cha>vacht^+(1-3)^: vacht^+(1-3)^*	3.57 (2.03 – 6.53)
	*Cha>vacht^+(1-7)^: Cha*	3.07 (1.75 – 5.68)
	*Cha>vacht^+(1-7)^: vacht^+(1-7)^*	4.03 (2.28 – 7.44)
		
(ii)	*Cha>vacht^Y49N(18)^: Cha*	>74.4
	*Cha>vacht^Y49N(18)^: vacht^Y49N(18)^*	>180.2
	*Cha>vacht^Y49N(19)^: Cha*	>74.4
	*Cha>vacht^Y49N(19)^: vacht^Y49N(19)^*	>252.9
	*Cha>vacht^Y49N(24)^: Cha*	>723
	*Cha>vacht^Y49N(24)^: vacht^Y49N(24)^*	>2607
		
(iii)	*Cha>vacht^E341K(5)^: Cha*	0.83 (0.55 – 1.26)
	*Cha>vacht^E341K(5)^: vacht^E341K(5)^*	2.8 (1.8 – 4.2)
	*Cha>vacht^E341K(7)^: Cha*	0.47 (0.31 – 0.71)
	*Cha>vacht^E341K(7)^: vacht^E341K(7)^*	1.3 (0.8 – 1.9)
	*Cha>vacht^E341K(9)^: Cha*	1.63 (0.97 – 2.74)
	*Cha>vacht^E341K(9)^: vacht^E341K(9)^*	5.29 (3.09 – 9.29)

Resistance factors are ratios of LD_50_ values obtained by fitting a regression analysis to the relationship between % mortality and log (dose); parallel regression was accepted as the model for all the data. All genotypes are described in the online methods.

Resistance factors for ectopic expression of the wild-type *vacht* transgene in two independent lines, as compared to the parental genotypes (*Cha* and *vacht* ^+(1−n)^).

Resistance factors for ectopic expression of the *vacht^Y49N^* transgene in three independent lines, as compared to the parental genotypes. Since no mortality was observed at the highest dose tested (1 mg.ml^−1^), resistance factors are the minimum expected based on estimates of LD50 that assume parallel dose response curves to those observed in (i).

(i) Resistance factors for ectopic expression of the *vacht^E341K^* transgene in three independent lines, as compared to the parental genotypes. No significant resistance was detected in the test genotypes compared to both of the parental controls.

### A High Affinity Binding Site for Spiroindolines in Insect Tissues and its Relationship to the Vesicular Acetylcholine Transporter

It remained a possibility that Spiroindolines did not act directly at VAChT, either because mutations in VAChT are able to compensate for other effects, or because changes to the transport activity of VAChT were able to reduce exposure (VAChT has a broad substrate specificity [25]). A complementary approach to the characterization of the target protein allowed us to address this possibility.

**Figure 9:**
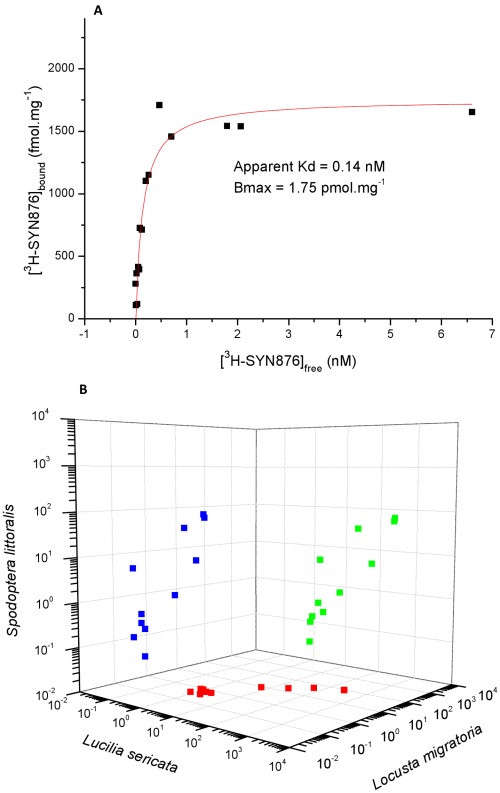
Binding of Spiroindolines in insect tissues. **A** - Saturation isotherm for the binding of [^3^H]-SYN876 in the blowfly *L. sericata*. Data from two independent experiments is combined and analyzed by fitting to a single site binding model (y = Bmax * x/(Kd+x)). The line represents the theoretical curve for the indicated values of Kd and Bmax. **B** - Two way correlation plots comparing the pharmacology of [^3^H]-SYN876 displacement between insect orders. *S. littoralis*,(Lepidoptera); *L. Sericata,* (Diptera); and *Locusta migratoria,* (Orthoptera). Data for the 24 analogues used is given in table S3.

**Figure 10:**
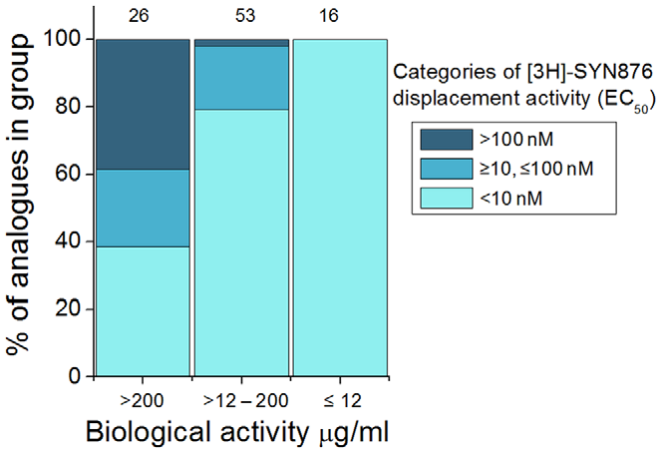
The relationship between insecticidal activity against lepidopteran larvae and potency in displacing [3H]-SYN876 from head membranes of *L. sericata*. The number of compounds in each category of biological activity is shown above the column. Structures of the analogues used and data for the correlation are given in tables S2 and S3. The category of biological activity was determined by the lowest EC_80_ against any of the species tested.

Conditions to allow measurement of saturable high affinity binding of [3H]-SYN876 to tissue homogenates from different insect species were established, revealing a very high affinity binding site at a concentration similar to that seen for the vesicular monoamine transporter in brain regions rich in dopaminergic neurons [26] (Figure 9a). Displacement assays demonstrated that the pharmacology of this binding site with respect to a variety of spiroindoline analogues was well conserved across insect orders (Figure 9b). Insecticidal spiroindolines generally had IC_50_’s in the low nM range in this assay, whereas a broad range of insecticides and drugs, diverse in terms of their chemical structure and known biochemical targets, were inactive at concentrations in the 1–10 µM range (table S4). These studies demonstrated the novelty and specificity of the binding site in the context of insecticide action. Correlation of the potency of spiroindoline analogues in the displacement assay with biological activity against lepidopteran larvae (Figure 10) indicated the relevance of this binding interaction to the insecticidal effect.

**Figure 11:**
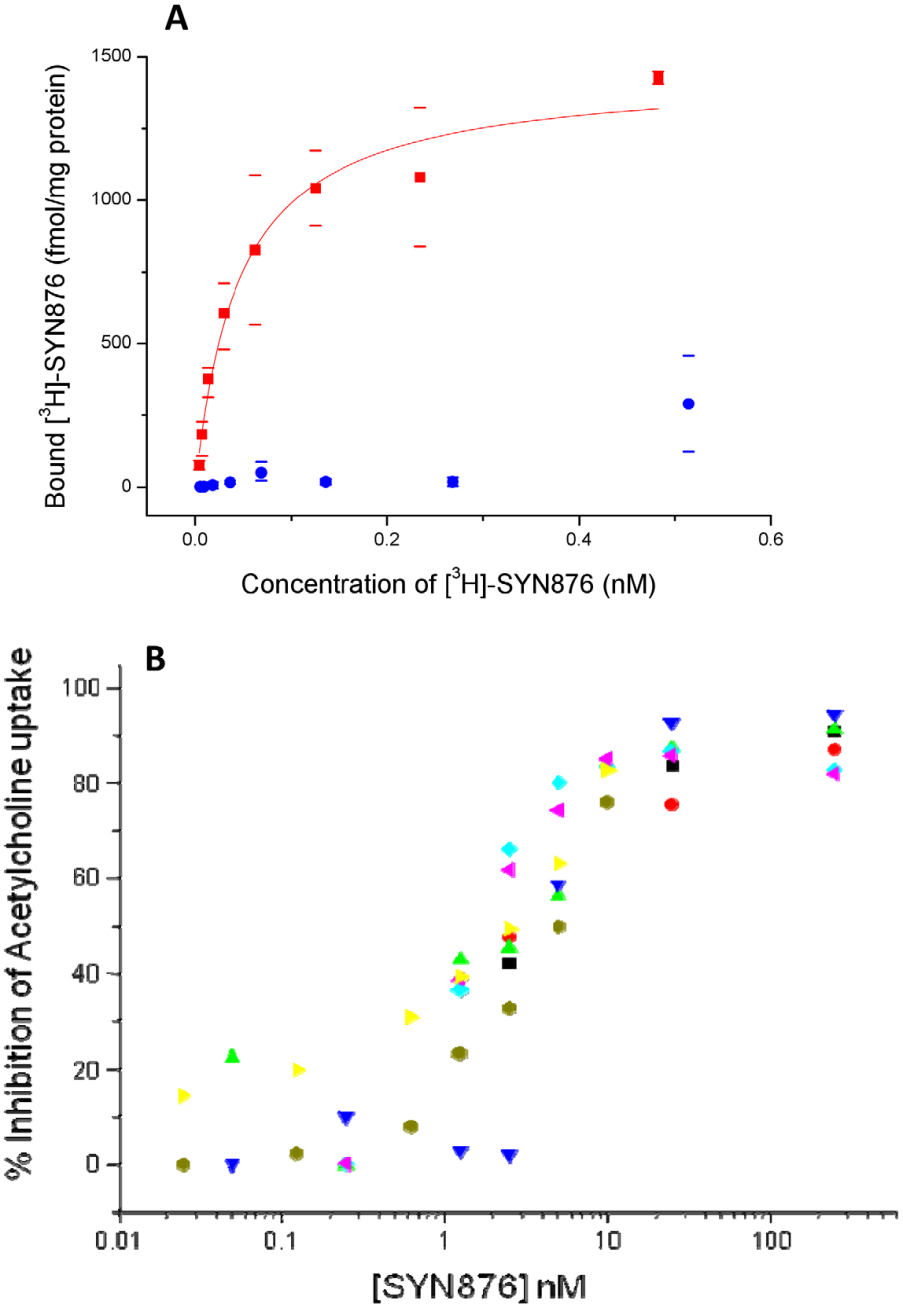
Binding properties and acetylcholine transport inhibition of SYN876 in PC12 cells expressing *D. melanogaster* VAChT. **A** – Binding of [^3^H]-SYN876 to membranes from PC12 cells. Red squares indicate average binding from cells transformed to express *D. melanogaster* VAChT, the line represents the theoretical curve for Kd = 0.04 nM and Bmax = 1.56 pmol.mg^−1^; blue circles indicate average binding from the same parent cell line transformed with the control vector; bars indicate maximum and minimum values (n = 4 below 0.05 nM [^3^H]-SYN876, 2 for data above). **B** - Inhibition of the acetylcholine transport activity of PNS from the cells transformed to express *D. melanogaster* VAChT. Colours indicate data from six independent experiments.

A binding site with very similar properties was produced in PC12 cells when transformed to express the D. melanogaster gene for VAChT (Figure 11), and it was shown that binding to this site and to the site in insect tissues was displaced by the known VAChT inhibitors vesamicol and aminobenzovesamicol (table S3). Thus it is clear that the Spiroindoline binding site in insect tissues corresponds to VAChT. Expression of D. melanogaster VAChT in PC12 cells allowed us to demonstrate that insecticidal spiroindolines are potent inhibitors of VAChT mediated transport of acetylcholine (Figure 11 and table S3).

## Discussion

As information is gathered on the activity of chemical libraries against a large number of drug targets, it becomes increasingly apparent that biologically active organic chemicals are rarely specific in their actions [27], [28]. Indeed, the ability of certain structural templates to interact with multiple receptors has been recognized since the 1980’s and led to the concept of privileged chemical structures [29]. For this reason it is not advisable to reach conclusions about the mechanism of a particular biological effect based solely on structural features of the ligand or its activity against isolated targets. In our own studies on isolated systems, early insecticidal spiroindolines showed activity against both the nicotinic acetylcholine receptor and voltage gated sodium channel at µM concentration (data not shown), targets that are unrelated to each other and to G-protein coupled receptors for which the spiroindoline scaffold is considered a privileged structure.

Pharmacological specificity is inversely related to dose, and so for very potent insecticides it is expected that the number of potential molecular interactions that could account for the biological effect will be small. However, the relationship between applied dose and concentration at the molecular target can often be confounded by clearance mechanisms, transport barriers, bioaccumulation, or a requirement for metabolic activation. So, to confidently assign any observed molecular interaction of a drug or agrochemical to its biological effect it is necessary to relate one to the other through experimental manipulation and correlation. The generation of resistance through mutagenesis is a powerful tool in this respect because it can quickly identify molecular changes that give rise to insensitivity at the whole organism level, sometimes indicating the identity or function of the target protein.

All of the mutations recovered and characterized in this study resulted in amino acid substitutions in conserved trans-membrane domains of the transporter (Figure 8). Apart from resistance to spiroindolines and, in one case, hypersensitivity to vesamicol, the mutants we recovered had no obvious phenotype as homozygotes. The genetic dominance of the mutations suggests that they directly impact the binding interaction with Spiroindolines, however, the distribution of the variant amino acids in a predicted protein structure (based on known structures of related bacterial proteins) does not allow all to form part of the same binding site [8]. Other available information on the function of amino acids at or close to the sites we identified indicates that many non-conservative substitutions result in impaired function, although none of the changes generated by site directed mutagenesis of the rat gene [30], [31], [32], or recovered in phenotypic screens of *C. elegans* [22], [33] are the same as reported here, so it is not possible to draw conclusions about the mechanism of resistance without further study.

Another approach to the identification of the relevant target protein is through the characterization of the highest affinity interactions of the ligand in tissues from the target organism. The very high affinity binding interaction of Spiroindolines in insect tissues is here linked to VAChT through its known pharmacology (sensitivity to vesamicol) and through the dependence of binding on the expression of VAChT in PC12 cells. It is linked to biological activity by correlation for a large number of Spiroindoline analogues. It seems that very potent inhibition of VAChT is a requirement for lethality, as is almost complete loss of function in genetic studies [22]. Although the mechanism of resistance has yet to be confirmed, it is clear from the studies presented here that Spiroindolines exert their most potent biological effects in insects and nematodes through inhibition of the transport activity of VAChT.

Insecticidal spiroindolines are structurally distinct from vesamicol and its analogues and so represent a novel class of ligand that should complement vesamicol in studies of the structure and function of VAChT [34]. Although vesamicol analogues compete with Spiroindolines for binding, the hypersensitivity of some Spiroindoline resistant *C. elegans* mutants to vesamicol (Figure **7**) indicates a different mode of interaction with the protein. Vesamicol is highly toxic to mammals [40], but is not insecticidal and to some degree this may be a consequence of selectivity at the protein level. Vesamicol binds with low nM affinity in vertebrate tissues [34], but binding could not be detected in insect tissues (data not shown), presumably because the affinity is too low, as indicated by the high IC_50_ for spiroindoline displacement (Table S4). The spiroindoline SYN876 has favourable acute oral toxicity in the rat, but is a potent insecticide. Studies of its interaction with VAChT from vertebrate species will be required to understand to what degree differential toxicity can be attributed to differences in binding, and such studies cold also inform strategies to design selectivity between target and non-target species.

Apart from their utility as insecticides and nematocides, Spiroindolines may also provide an alternative to vesamicol analogues for the development of reagents to image cholinergic neurons in the human brain by positron emission tomography, potentially useful in the diagnosis and study of a number of neurodegenerative and psychiatric conditions. Currently available reagents based on the structure of vesamicol have a number of limitations for this purpose [35].

## Materials and Methods

### Materials

Synthetic and analytical methods for Spiroindolines are described online, as are the sources of other chemicals, reagents and biological materials.

### Preparation and Assay of Fractions from Insect Tissues and PC12 Cells

Methods for membrane preparation and assays for radioligand binding and vesicular acetylcholine uptake were adapted from those previously described for insects [36] and PC12 cells [37]. PC12 cells expressing the *D. melanogaster vacht* gene were generated using the Invitrogen™ Gateway® cloning technology. The *D. melanogaster* gene was cloned by high fidelity PCR from a cDNA library prepared from adult flies.

### Production of Spiroindoline Resistant Strains of *C. elegans* and *D. melanogaster*

Resistant *C. elegans* mutants were selected by exposure to SYN351 (5 µg.ml^−1^) from the F2 generation following EMS mutagenesis using standard methods [38]. Details of mapping are given in the online methods section. Variant *unc-17* sequences were obtained by PCR cloning from mixed stage *C. elegans* cultures (ENA accession numbers: FR852384 (*cb28*), FR852389 (*cb29*), FR852385 (*cb30*), FR852386 (*cb31*), FR852387 (*cb32*) and FR852388, (*cb34*)).

*D. melanogaster* was transformed to over express wild type and variant vacht using the GAL4-mediated binary expression system [24] as modified by Griswold et al [39]. The transgenic lines were crossed to the cha-GAL4 and the elav-GAL4 driver lines to give tissue specific transgene expression. Genotypes are fully described in the online methods section.

### Bioassays

Insects and nematodes were exposed to test chemicals both through the diet and by contact. Chemicals were introduced in a solvent that was also present in the controls and that alone had no effect on survival. Effects were assessed after 3–6 days of exposure by manual observation and used to generate dose response curves from which measures of potency were derived. Acute toxicity in the rat was assessed 7 days after a single oral dose.

Methods are described in detail in Text S1.

## Supporting Information

Text S1 **Supplementary methods and validation.** This document contains detailed descriptions of the methods used and additional results supporting experimental interpretation in the main text.
(DOC)

Table S1 **Insecticidal activity of a selected set of spiroindoline compounds against lepidopteran larvae.** Activity is given as the concentration giving 80% mortality (EC_80_ (µg.ml^-1^)).Use of < and ≤ indicate that the operator judges the true EC80 to be well below and a little below, respectively, the given concentration but above the next lowest concentration tested.
(DOC)

Table S2 **Structures and biological activities of spiroindoline analogues.** Activity is given as the concentration giving 80% mortality (EC_80_ (µg.ml^-1^)).Use of < and ≤ indicate that the operator judges the true EC80 to be well below and a little below, respectively, the given concentration but above the next lowest concentration tested.
(DOC)

Table S3 **Potency of spiroindoline analogues for displacement of Spiroindoline binding and inhibition of vesicular acetylcholine uptake.** Columns headed with the insect species names show data for displacement of [^3^H]-SYN876 binding. Acetylcholine uptake was measured using a fraction isolated from PC12 cells expressing Drosophila VAChT. Displacement and inhibition assays are described in the Text S1. Missing values were not determined. Some values are ranges or approximations (∼) based on a limited number of concentrations tested; others were determined by curve fitting as described in Text S1. Compound numbers refer to the structures in Table S2.
(DOC)

Table S4 **Pharmacological selectivity of the Spiroindoline binding site in** ***L. Sericata.*** Compounds were assayed for [3H]-SYN876 displacement as described in Text S1. Compounds were scored as inactive if they gave less that 20% displacement at the highest test concentration (nominally 10 µM).
(DOC)
