# Supplementary material for: Spiroindolines Identify the Vesicular Acetylcholine Transporter as a Novel Target for Insecticide Action
Source: PLoS One. 2012 May 1;7(5):e34712. doi: 10.1371/journal.pone.0034712 (PMC3341389; doi:10.1371/journal.pone.0034712)
Supplement: Text S1 — Supplementary methods and validation. This document contains detailed descriptions of the methods used and additional results supporting experimental interpretation in the main text. (DOC) [file pone.0034712.s001.doc]

## Supplementary methods and validation.

Biological materials.
*C. elegans* strains utilized in this study included N2 (wildtype), PR1152 [cha-1(p1152) IV], MT1001 [lin-1(e1777) IV], CB1166 [dpy-4(e1166) IV], BC23 [unc-22(s7) IV], EG1000 [dpy 5(e61) I; rol-6(e187) II; lon-1(e1820) III], and EG1020 [bli-6(sc16) IV; dpy-11(e224) V; lon-2(e678) X]. Some strains were obtained from the Caenorhabditis Genetic Center. *D. melanogaster* strains cha-GAL4 *(w*; P{w[+mC]=Cha-GAL4.W}19B)* and elav-GAL4 *(P{w[+mW.hs]=GawB}elav[C155])*, were obtained from the Bloomington Drosophila Stock Center, Department of Biology, Indiana University , 1001 E. 3rd St., Bloomington, IN 47405-3700, USA; other transgenic lines were generated in house. *L. sericata* were obtained from Blades Biological Ltd, Cowden, Edenbridge, Kent, TN8 7DX, as pupae and adults maintained on sucrose solution for up to 2 days after emergence. Other insects used for membrane preparations and laboratory based biological assays were from cultures maintained at Syngenta facilities in Stein, Switzerland. PC12 cells were obtained from ATCC (catalogue number CRL 1721) via LGC Standards, Queens Road, Teddington, Middlesex, TW11 0LY.

Preparation of membrane fractions from insect tissues and PC12 cells.
Membranes from insect tissues were prepared essentially as described [36]. Heads of adult *L. sericata*, heads of fourth or fifth instar larvae of *S. littoralis*, or sub-oesophageal ganglia of *L. migratoria*, were homogenized in 0.32M Sucrose, 50mM Tris-HCl, 1mM EDTA, 1µM Leupeptin, 1µM Pepstatin, 1mM PMSF, pH7.4, at the ratio 1 g of tissue to 3 ml of buffer. The homogenate was filtered through muslin, centrifuged at 6,500 x g for 10 minutes to remove cell debris and then at 190,000g for 60 minutes. The pellets were re-suspended to a protein concentration of at least 10 mg.ml-1 in 0.12M NaCl, 50mM Tris-HCl, 100µM EDTA, pH 7.4, used immediately or stored frozen in liquid nitrogen.

PC12 cells were grown to a high density in 175cm tissue culture flasks with F-12K nutrient mixture (Invitrogen) supplemented with 10% horse serum and 5% foetal calf serum (FCS). A post-nuclear supernatant (PNS) was prepared from the cells essentially as described by Ojeda *et al* [30]. The growth medium was removed, the cells dissociated by incubating in 10 ml CDS (Sigma C154) and washed with PBS. The cell pellet derived from 14 flasks was re-suspended in 3.5 ml of homogenization buffer (10 mM N-(2-hydroxyethyl)piperazine-N‘-2-ethanesulfonic acid (HEPES) adjusted to pH 7.4 with KOH, 0.32 M sucrose, 1mM PMSF, 12.6uM Leupeptin, 5ug/ml Aprotinin and 50uM Eserine), and most of the cells disrupted in a Potter-Elvehjem homogenizer. Remaining cells and cell debris was separated by centrifugation at 800g for 10 mins, the pellet re-homogenized in a further 3.5 ml of buffer and centrifuged again. The supernatants were combined and used immediately for assay of vesicular acetylcholine uptake. This fraction was also used for assay of ligand binding activity, in which case it could be stored frozen in liquid nitrogen. All operations were conducted on ice or at 4ºC.

Radio-ligand binding and displacement assays.
[3H]-SYN876 binding to membranes from insect tissues or PC12 cells was measured by the rapid filtration method [41]. For characterization of ligand affinity and binding site concentration in head membranes of the blowfly *Lucilia sericata*, increasing concentrations of [3H]-SYN876 were incubated in glass tubes with 200 µg of membrane protein in 3 ml assay buffer (50mM TRIS, 120mM NaCl, 100uM EDTA pH 7.4) for 1 hr at room temperature with shaking. The binding was stopped by the addition of 3ml ice cold assay buffer and membranes were separated by rapid filtration through glass micro-fibre filters (Whatman GF/C), pre-wetted with the same buffer supplemented with 0.25% (w/v) polyethyleneimine and 0.15% (w/v) Triton X-100. The membranes were washed on the filter with buffer supplemented with 0.05% (w/v) Triton X-100. Bound [3H]-SYN876 was determined by scintillation counting and corrected for non-specific binding, the free concentration was determined by subtraction from the total concentration. Non-specific binding was determined by duplicate incubations in the presence of 1 µM SYN876 and was <10% of total binding. The final (total) concentration of [3H]-SYN876 in the incubation mix was determined by sampling and scintillation counting to correct for errors due to adsorption during handling. [3H]-SYN876 was added to the incubation tubes as an ethanolic solution and unlabelled SYN876 as a solution in DMSO. The final concentration of ethanol and DMSO was adjusted to 0.25% and 0.625% respectively in all tubes. Binding to the PNS prepared from PC12 cells was measured in the same way except that 30 µg of protein was used and the final incubation volume was 8 ml. Saturation data was analyzed to obtain the apparent dissociation constant (Kd) and binding site concentration (Bmax) by fitting to the one site binding model (y = Bmax * x / (Kd + x) where y and x are bound and free ligand concentrations respectively) using the OriginPro 8 software package (Origin lab Corporation).

Competition binding assays were performed in microtitre plates in a final volume of 200 µl containing 10 µg insect membrane protein. [3H]-SYN876 (to give 1 nM final concentration) and various concentrations of competing ligands were added in 5 µl DMSO, which was also present in the controls. Competing ligands were generally tested at 12 concentrations in duplicate spanning the range 1 pM – 1 µM. Filtration and washing used a Tomtec cell harvester. Data was analyzed to obtain the IC50 value (50% displacement of [3H]-SYN876) by fitting the means to the one site competition model using the OriginPro 8 software package.

Protein concentration was determined using the Bradford reagent.

Chemical mutagenesis and mapping of resistance mutations in C. elegans.
Spiroindoline-resistant mutants were recovered by SYN351 (5 μg.ml-1) selection of the F2 generation following EMS mutagenesis of the N2 wild-type strain by standard methods [38]. Ten mutants exhibiting heritable SYN351 resistance were recovered from ~1.7 x 10^6^ mutagenized genomes, representing at least six independent mutations. All recovered SYN351-resistance alleles were genetically dominant. Seven mutants were each outcrossed four times to the parental N2 strain prior to further characterization. The mutants exhibit no overt phenotypes in the absence of SYN351. When exposed to SYN351, the seven outcrossed alleles fall into two phenotypic classes: (1) “weak” resistance, represented by alleles *cb28*, *cb29*, and *cb31* (strains LL1628, LL1629, and LL1631, respectively), viable in the presence of 1 μg/ml SYN351, but with uncoordinated movement and variable growth rate; (2) “strong” resistance, represented by alleles *cb30*, *cb32*, *cb33*, and *cb34* (strains LL1630, LL1632, LL1633, and LL1634), resistant to >1 μg.ml-1 SYN351, with coordinated movement and consistently strong growth in the presence of 1 μg.ml-1 SYN351. All seven of these alleles mapped to linkage group (LG) IV (i.e., failed to segregate independently from the LGIV marker *bli-6(sc16)* following a cross with the strain EG1020).

Initial two-factor genetic mapping placed the SN120-resistance alleles *cb31* and *cb34* in a broad region in the left center of LGIV, using the markers *dpy-4(e1166)* and *lin-1(e1777)*. From crosses of each marker to each of the mutants, F2 progeny homozygous for the marker were tested for resistance to SYN351 that would indicate a recombination event had occurred. From the *dpy-4* crosses, of 6 of 29 Dpy F2 progeny scored carried *cb34*, and 7 of 26 Dpy F2 progeny carried *cb31*. From the *lin-1* crosses, 3 of 8 Lin F2 progeny carried *cb34*, and 4 of 25Lin F2 progeny carried *cb31*.

Based on the two-factor mapping data, the doubly marked strain *lin-1(e1777) unc-22(s7)* was constructed for use in three-factor mapping. To refine the placement of SYN351-resistance alleles on LGIV, *cb31* and *cb34* were each crossed with the *lin-1 unc-22* strain and individual Lin NonUnc and Unc NonLin recombinants were collected from the F2 cross progeny. These F2 recombinants were tested for the presence of SYN351 resistance. For *cb31*, 14 of 25 Unc NonLin and 15 of 24 Lin NonUnc F2 progeny were resistant to SYN351. For *cb34*, 14 of 25 Unc NonLin and 9 of 23 Lin NonUnc F2 progeny were resistant to SYN351. The modified Wald equation (for small experimental N) was used to determine 95% confidence intervals for the recombinant ratios [42]. These resulting recombinant ratio intervals were used to determine map distances between the SYN351-resistance alleles and the selected flanking markers, with the corresponding genetic confidence intervals, yielding calculated genetic map positions. For *cb31*, the calculated genetic map position was -2.2 ± 2.5 map units based on *cb31* Unc NonLin recombinants, or 0 ± 2.5 map units based on *cb31* Lin NonUnc recombinants. For *cb34*, the calculated position was -2.2 ± 2.5 map units based on *cb34* Unc NonLin recombinants, or -2.8 ± 2.5 map units based on *cb34* Lin NonUnc recombinants. Thus these results placed both *cb31* and *cb34* in overlapping 5 map unit genetic intervals, suggesting that they could affect the same gene, though the genetic dominance of SYN351 resistance precluded complementation testing.

The genetic intervals to which *cb31* and *cb34* were mapped spanned the genetic map position of the complex locus encoding VAChT (*unc-17*) and ChAT (*cha-1*) [23, 43]. cDNA sequences spanning the protein-coding regions of each of these genes were amplified by reverse transcription-polymerase chain reaction (RT-PCR) from each of six independent SYN351-resistant mutant lines, using a high fidelity polymerase (Platinum® Pfx, Invitrogen). The primers used for amplification were:
unc-17.f1 TTT TCC GCA TCT CTT GTT CA
(Forward primer designed in the 5’ untranslated exon common to both *unc-17* and *cha-1*)

unc-17.r1 CAC CAA GGT TTC GAT TCT TTT
(Reverse primer designed in the 3’ UTR of spliced *unc-17*)

cha-1.f1 TCT TGA CAC TTG GTT AGT GTT TTA GC
(Forward primer designed in the 5’ UTR of spliced *cha-1*)

cha-1.r1 TTC TGA GCA CTC AAC TAC ATG C
(Reverse primer designed in the 3’ UTR of spliced *cha-1*)

The gel-purified RT-PCR amplification products were sequenced directly, revealing mutations in *unc-17* coding sequences for all six mutants that predicted four amino acid substitutions (Figure 8). Variant *unc-17* coding sequences have been submitted to the European Nucleotide Archive (accession numbers: FR852384 (*cb28*), FR852389 (*cb29*), FR852385 (*cb30*), FR852386 (*cb31*), FR852387 (*cb32*) and FR852388, (*cb34*)).

Cloning of Drosophila melanogaster vacht and assembly of vectors for transformation.
RNA was extracted from 40 wild type Canton-S *D. melanogaster* flies using the TRIzol® Reagent yielding 10ug of RNA at 1µg.µl-1 which was used to construct three independent cDNA libraries. DNA representing *D. melanogaster* *vacht* was amplified by high fidelity polymerase chain reaction (with Pfu polymerase, Agilent Technologies) using the following primers:

Drosophila 5' ATGGCCTCATTCCAAATACCTGTTATC
Drosophila 3'XhoI CTCGAGTTAAAATCCTTGCCTAAAGGGATTCG

The amplified band representing the full length *vacht* coding sequence was purified and ligated with the Gateway® entry vector pENTR1A linearized by digestion with XmnI and XhoI (Qiagen gel extraction and PCR purification kits were used for purification, Merck Clonables™ Ligation kit for ligation). The ligation product was transformed into Top10 *E-coli* cells, colonies screened using the PCR primers and four of the many positives were mini-prepped. Sequencing of the insert in one clone (pENTR™ 1A/VAChT 15) confirmed the database sequence (FlyBase ID FBpp0083086) except for three known polymorphisms T97S, A160V, L272M and a previously unknown polymorphism (a triplet codon deletion in a run of 13 glutamine coding triplets close to the C terminus). This polymorphism was confirmed by sequencing of PCR products from the other cDNA libraries. This sequence is referred to elsewhere in this publication as the wild type sequence.

Site directed mutagenesis was carried out on pENTR1A VAChT 15 to produce the Y49N and E341K variants using the Stratagene Quikchange XL kit and the following primers;

Y49N-FOR CTGGACAACATGCTGAACATGGTGATAGTAC
Y49N-REV GTACTATCACCATGTTCAGCATGTTGTCCAG
E341K-FOR GGATTGGCCCTGAAGGGCTTCTCCTGCTTC
E341K-REV GAAGCAGGAGAAGCCCTTCAGGGCCAATCC

DpnI digestion was used to remove the parental DNA, leaving only plasmid containing the desired mutation. These were transformed into Top10 E.coli cells, grown up and mini-prepped. Sequencing confirmed that only the desired changes had occurred.

The ORFs (wild type, Y49N and E341K variants) were also cloned downstream of the UAS promoter into the pB-UGateway w+ vector [39]. This enables expression of the transgenes in Drosophila under the control of a GAL4-mediated binary expression system [24].

Production of a stable PC12 cell line expressing D. melanogaster VAChT.
The wild type vacht sequence from pENTR™ 1A/VAChT 15 was cloned into the pcDNA™3.2/V5-DEST Gateway® vector using the LR recombination reaction, and transfected into PC-12 cells following protocols provded by Invitrogen™. The presence of the insert was checked by restriction analysis with NdeI/XhoI double digests. For transfection, 2.5x10^5^ cells in 2 ml F12K medium containing 10% FCS were seeded into 35mm wells. After 20 hours the medium was replaced with Opti-MEM® containing 5 µg vector DNA complexed with Lipofectamine™, incubated at 37°C for 4.5 hrs and then replaced with 2.5ml F12K containing 10% horse serum and 5% FCS. After 24 hrs the cells were transferred to collagen coated flasks. Selection with 0.6 mg.ml-1 G418 was initiated after a further 24 hrs and maintained for 4 weeks with twice weekly changes of growth medium. Subsequently, cells expressing *D. Melanogaster* VAChT were cloned by serial dilution and maintained in the presence of 0.3 mg.ml-1 G418. Transgene expression was assessed by immunocytochemistry using rabbit anti -*D. Melanogaster* VAChT polyclonal antiserum [44] as the primary antibody.

Generation of transgenic flies.
Transformation was performed by microinjection of these constructs together with a piggyBac transposase containing plasmid [39] into the w1118 Drosophila melanogaster strain [45]. Multiple lines were obtained for each construct: lines carrying single copies of the wild-type *vacht* transgene are referred to as *vacht+(1-3)* and *vacht+(1-7)*; lines carrying single copies of the Y49N variant transgene are referred to as *vachtY49N(18)*, *vachtY49N(19*) and *vachtY49N(24)*; and lines carrying single copies of the E341K variant transgene are referred to as *vachtE341K(5)*, *vachtE341K(7)* and *vachtE341K(9)*.

To drive expression of these transgenes, the *cha*-GAL4 driver and the *elav*-GAL4 drivers were crossed to these transgenic lines. The resulting genotypes referred to in Table 1 are as follows: *cha*-GAL4 driven expression of the wild-type vacht transgene in two independent lines is referred to by *Cha>vacht+(1-3)* and *Cha>vacht+(1-7)*; *cha*-GAL4 driven expression of the Y49N variant transgene in three independent lines is referred to by *Cha>vachtY49N(18)*, *Cha>vachtY49N(19)* and *Cha>vachtY49N(24)*; and *cha*-GAL4 driven expression of the E341K variant transgene in three independent lines is referred to by *Cha>vachtE341K(5)*, *Cha>vachtE341K(7)* and *Cha>vachtE341K(9)*. The *cha*-GAL4 driver line itself is referred to as *Cha* in Table 1.

Measurement of acetylcholine uptake activity in PC12 cells.
The method used followed that described by Veroqui *et al* [37]. The post nuclear supernatant (PNS) was freshly prepared as described in the section ‘Preparation of membrane fractions from insect tissues and PC12 cells’. The assay was conducted in 96 well microtitre plates. Test chemicals were introduced as solutions in 1 µl of dimethyl sulphoxide (also present in the controls) to which was added 150 µl PNS which had been diluted to 2 mg protein.ml-1 in uptake buffer (110 mM potassium tartrate, 20 mM HEPES, 50µM eserine, pH 7.4) and incubated for 10 minutes at 37°C. The uptake reaction was started by the addition of Mg2+ ATP and acetylcholine in 50 µl of uptake buffer to give final concentrations of 10 mM and 1.5 mM respectively. The acetylcholine was two parts unlabelled acetylcholine chloride and one part [3H]- acetylcholine iodide (NEN, 2.8GBq.mmol-1). The reaction mixture was incubated for 2 hrs. at 37°C then rapidly filtered through a glass fibre glass micro-fibre filter (Whatman GF/C), pre-wetted with ice cold 0. 5% (w/v) polyethyleneimine. The captured membranes were rapidly washed on the filter with ice cold uptake buffer and the remaining radioactivity measured by scintillation counting. Chemical effects were calculated as percentage inhibition of the untreated control. Radioactivity retained on the filters was shown to be dependent on the presence of ATP and sensitive to both aminobenzovesamicol (ABV) and bafilomycin [average counts recovered ± SD; + ATP = 474 ± 144, - ATP = 196 ± 53, n = 20; + 0.8 µM bafilomycin 96± 10, + 1 µM ABV = 110 ± 21].

*C. elegans* bioassays.
*C. elegans* bioassays were carried out by adding compounds to the agar culture medium. For each experiment the indicated strains were assayed in parallel for comparison. Compounds were added as solutions in dimethyl sulphoxide (DMSO) or propan-2-ol:DMSO:water (50:10:40 v/v), and the final solvent concentrations in all assays was 1% (DMSO) or 3% and 0.6% (propan-2-ol and DMSO respectively). All assays were performed in triplicate. Synchronously staged L1 hermaphrodite larvae were placed in the assay wells (average 22 animals per assay; exact number determined by counting). Animals were monitored for effects on nematode movement and viability. After three days, nematode survival was assessed in each well by counting the number of adults. Untreated control cultures routinely contain >90% gravid adults at the time of scoring.

*D. melanogaster* Bioassays.
Bioassays were set up as follows to expose the adult insects to test compounds by contact and feeding: a 5% sucrose:1% agar mix was prepared and 0.75 mls poured into each well of a 24-well plate, and allowed to set. The test compound was dissolved in a suitable ethanol-acetone based solvent system to the dilutions required, to a maximum of 1000 μg.ml-1 after which the compound was insoluble. 20 µl of a single dilution was spread uniformly on the surface of the sucrose-agar base in a well. A dose range was created as a typical plate contained 5 different concentrations of insecticide in consecutive wells and one control (formulation solution only).

0-3 day old adult flies of the appropriate genotype were sorted and 10 flies were placed per well, with at least 3 replicates per dose. The plates were sealed with a breatheable film (BEM1, Web Scientific), and incubated under normal culture conditions (25°C, 70-75% humidity). Each experiment was repeated at least 3 times. Mortality and any other visible effects were assessed up to 6 days after treatment. Comparisons between different genotypes were carried out on data from assessments that were done at the same time and by the same individual assessor. Mortality data were subjected to logit analysis [46]. Each genotype/compound combination was analyzed separately, fitting parallel dose response lines to all the genotypes within each test, in order to generate LD50 for each genotype. Resistance Factors were calculated by dividing the first strain (test cross) LD50 by the second strain (parental line) LD50. The estimated resistance factor was accompanied by a 95% confidence interval.

Other insect bioassays.
The insecticidal activity of Compound **1** (figure 1 main paper)was measured in 96 well microtitre plates.  The insect species, lifestage and host plant or media used were; *Plutella xylostella* eggs on leaf discs of Chinese cabbage;  *Heliothis virescens* eggs on cotton leaf discs, *Drosophila melanogaster* eggs on artificial diet, and *Aedes aegypti* first instar larvae in liquid media.  Compound **1** was applied to the wells after insect infestation as a 1 mg.ml-1 solution in 5 µl of a suitable solvent.  Mortality was assessed relative to solvent only controls after 6 days. Compound **1** showed no effect against *Aedes aegypti* but ≥80% mortality by visual assessment against all other species.

The insecticidal activity of compounds in Table S1 and Table S2 was measured as follows. For *S. littoralis*, cotton leaf discs were placed on agar (0.5 ml) in a 24-well microtiter plate and sprayed with 15 µl of test solutions (water based containing 2%DMSO and <1% surfactant) at concentrations of 200, 50, 12, 3, 0.8 and 0.2 µg.ml-1. After drying, the leaf discs were infested with 5 first instar larvae and scored for insecticidal activity after 3 days. For *H. virescens,*eggs (0-24 h old) were placed in 24-well microtiter plate on artificial diet (0.5 ml) and treated with test solutions (40 µl) at the same concentrations by pipetting. They were scored for insecticidal activity after 4 days. For *P. xylostella*, wells of a 24-well microtiter plate containing artificial diet (0.5 ml) were treated with test solutions (50 µl ) at the same concentrations by pipetting. After drying, the diet was infested with second instar larvae (7-12 per well) which were scored for insecticidal activity after 6 days. Insecticidal activity was visually assessed as failure of eggs to hatch, or of larvae to respond, feed and develop normally compared to controls. An effective concentration to give 80% activity (EC80) was derived by the assessor relative to the test concentrations.

Field efficacy of SYN876 (Figure 3).
For *Plutella xylostella*, (diamond-back moth) on brassicae (cabbage, cauliflower or broccoli) two spray applications were made with an interval of 7 days. Assessments (2-5) were made between 3 days after the first application and 10 days after the second by counting live larvae on 5 randomly selected plants in each of 3 replicate plots from treatment and control groups. The value for each point represents the average across these assessments.

For *Spodoptera littoralis*, (Egyptian cotton leafworm) on cotton a single application was made. Leaves were collected from 10 randomly selected plants in duplicate plots from treatment and control groups immediately after application, and 3 and 6 days after application. Leaves were infested in the laboratory and assessments made 6 days later by counting live larvae. The value for each point represents the average of these assessments.

For *Cydia pomonella*, (codling moth) on apple trees. Four assessments of larval control were made between 1 and 22 days after a single spray application. The value for each point represents the average of these assessments.

Assessment of acute toxicity in the rat.
A single dose of SYN876 was administered by gavage as a suspension in 0.5% w/v aqueous carboxymethylcellulose at 10 ml.kg-1 bodyweight to 2, 9 week old Female Sprague Dawley rats. The dose of SYN876 was 200 mg.kg-1. No mortality was observed during the 7 day assessment period. Testing was conducted by SafePharm Laboratories Ltd, London Road, Shardlow, DE72 2GD, UK.

Chemicals and reagents.
Chemical and analytical methods for Spiroindolines are given in supplementary materials. (±)-Vesamicol hydrochloride was purchased from Sigma-Aldrich® , (±)-4-Aminobenzovesamicol was purchased from American Radiolabelled Chemicals Inc., 101 ARC Drive . Saint Louis, MO 63146 USA. Other chemicals listed in Table S4 were from the Syngneta collection or were purchased from Dr. Ehrenstorfer GmbH or Sigma-Aldrich®. General laboratory reagents were from Sigma-Aldrich® unless otherwise specified. Reagents for gene cloning and transformation were from Invitrogen™ unless otherwise stated. Anti-Drosophila VAChT Polyclonal Antiserum from rabbit was a gift from Prof. Toshihiro Kitamoto, Department of Anaesthesia, University of Iowa.

Chemical synthesis.

**General synthetic method (path a): synthesis of 1-(2-chloropyridin-4-yl)carbonyl-1’-[*trans*-3-(4-chlorophenyl)allyl]-5-chlorospiro[indoline-3,4’-piperidine] 2**

Step 1: 4-methoxymethylenepiperidine-1-carboxylic acid *tert*-butyl ester

Potassium *tert*-butoxide (154 g, 1.37 mol) was added in portions to a stirred solution of methoxymethyltriphenylphosphonium chloride (570 g, 1.66 mol) in anhydrous THF (2000 ml) under an atmosphere of nitrogen at 4ºC. A vivid orange colour was noted and the reaction was left as such for 1 h. 4-Oxopiperidine-1-carboxylic acid *tert*-butyl ester (300 g, 1.51 mol) was added slowly not letting the temperature rise above 10 ºC and the mixture was then allowed to warm to room temperature overnight. The reaction mixture was poured onto water, extracted three times with ethyl acetate and the combined organics were washed with brine, dried over anhydrous sodium sulphate and concentrated *in vacuo* to yield a brown oil. Flash chromatography [SiO2; hexane, then ethyl acetate-hexane (10:90)] yielded the title product (284 g, 84%). 1H NMR (400 MHz, CDCl­3) 1.5 (9H, m), 2.0-2.2 (m, 4H), 3.4 (m, 4H), 3.5 (s, 3H), 5.9 (s, 1H). MS (ES+) 228 (M+H+), 172 (M-ibutene+H+)

Step 2: 5-chlorospiro[indoline-3,4’-piperidine]-1’-carboxylic acid *tert*-butyl ester

Trifluoroacetic acid (6 ml) was added to a stirred solution of 4-methoxymethylene-piperidine-1-carboxylic acid *tert*-butyl ester (5 g, 2 mmol), 4-chlorophenylhydrazine hydrochloride (3.95 g, 22 mmol) and ethanol (5 ml) in chloroform (500 ml) at 4 ºC under an atmosphere of nitrogen. The mixture was then stirred at 50ºC overnight, turning a dark green colour. The reaction was quenched with concentrated ammonia solution (250 ml) in ice water (500 ml), the organic layer turning orange. The organic layer was separated and the aqueous was further extracted twice with dichloromethane. The combined organics were washed with brine, dried over anhydrous sodium sulphate and concentrated *in vacuo* to yield the crude imine 5-chlorospiro[3*H*-indole-3,4’-piperidine]-1’-carboxylic acid *tert*-butyl ester as an orange foam. 1H NMR (400 MHz, CDCl­3) 1.5 (9H, m), 1.70 (m, 2H), 1.85 (m, 2H), 3.50 (m, 2H), 4.05 (m, 2H), 7.35 (m, 2H), 7.60 (s, 1H), 8.35 (s, 1H). MS (ES+) 321/323 (M+H+), 265/267 (M-ibutene+H+), 221/223 (M-Boc+H+). Sodium borohydride (2.86 g, 75 mmol) was added to a stirred solution of crude imine in absolute ethanol (120 ml) under an atmosphere of nitrogen. The reaction was stirred for 15 min and left to stand overnight. The mixture was concentrated *in vacuo* and the residue re-dissolved in dichloromethane (400 ml). The organics were washed with water and brine, dried over anhydrous sodium sulphate and concentrated *in vacuo* to yield a brown solid. Flash chromatography [SiO­2: ethyl acetate-hexane-triethylamine (25:75:1)] yielded the title product (2.98 , 42%, over both steps). M.p. 165-166 °C. 1H NMR (400 MHz, CDCl­3) 1.5 (9H, s), 1.70 (m, 4H), 2.9 (m, 2H), 3.50 (s, 2H), 3.75 (br s, 1H), 4.05 (m, 2H), 6.55 (d, J = 6Hz, 1H), 7.00 (m, 2H). MS (ES+) 323/325 (M+H+), 267/269 (M-ibutene+H+), 223/225 (M-Boc+H+).

Step 3: 1-(2-Chloropyridin-4-yl)carbonyl-5-chlorospiro[indoline-3,4’-piperidine]- 1’-carboxylic acid *tert*-butyl ester

To a solution of 5-chlorospiro[indoline-3,4’-piperidine]-1’-carboxylic acid *tert*-butyl ester (2 g, 6.2 mmol) and triethylamine (2 ml) in dichloromethane (50 ml) at 0°C was added 2-chloroisonicotinoyl chloride (prepared from 2-chloroisonicotinic acid (1.26 g, 8 mmol) and thionyl chloride (20 ml)) in dichloromethane (20 ml). The reaction mixture was stirred at room temperature for 2 hours then poured into 1M aqueous sodium bicarbonate. The organic layer was separated, the aqueous layer was extracted three times with dichloromethane, the combined organic layers were dried over magnesium sulfate then concentrated *in vacuo*. The residue was purified by column chromatography (hexane:ethyl acetate 50:50 +1% Et3N) to afford the title product (2.38 g, 94%). M.p. 231°C. MS (ES+) 462/464 (M+H+); 1H NMR (400 MHz, CDCl­3) 1.50 (s, 9H), 1.60-1.80 (m, 4H), 2.80 (m, 2H), 3.90 (m, 2H), 4.08 (s, 2H), 7.16 (s, 1H), 7.20 (m, 1H), 7.30 (d, J = 6Hz, 1H), 7.44 (s, 1H), 8.00-8.20 (br s, 1H), 8.54 (d, J = 6Hz, 1H).

Step 4: 1-(2-Chloropyridin-4-yl)carbonyl-5-chlorospiro[indoline-3,4’-piperidine] trifluoroacetic acid salt

1-(2-Chloropyridin-4-yl)carbonyl-5-chlorospiro[indoline-3,4’-piperidine]- 1’-carboxylic acid tert-butyl ester (2.15 g, 4.65 mmol) was dissolved in dichloromethane (50 ml) and trifluoroacetic acid (30 ml) was added slowly. The mixture was left to stand for 15 min, then the solvents were evaporated to leave a dark brown residues. Ether (100 ml) was added and the residue triturated to precipitate a yellow solid. The precipitate was collected by filtration with a top pressure of nitrogen and samples dried in vacuo at 40 °C. 1-(2-Chloropyridin-4-yl)carbonyl-5-chlorospiro[indoline-3,4’-piperidine] trifluoroacetic acid salt (2.28g, 96%). M.p. 236°C. MS (ES+) 362/363/364/365 (M+H+); 1H NMR (400 MHz, CDCl­3) 1.80 (m, 2H), 1.90 (m, 2H), 2.90 (m, 2H), 3.25 (m, 2H), 3.98 (s, 2H), 7.25 (s, 1H), 7.32 (br s, 1H), 7.53 (d, J = 7Hz, 1H), 7.64 (s, 1H), 8.10 (br s, 1H), 8.56 (d, J = 7Hz, 1H), 8.90 (br s, 2H).

Step 5: 1-(2-Chloropyridin-4-yl)carbonyl-1’-[*trans*-3-(4-chlorophenyl)allyl]-5-chlorospiro[indoline-3,4’-piperidine]

1-(2-Chloropyridin-4-yl)carbonyl-5-chlorospiro[indoline-3,4’-piperidine] trifluoroacetic acid salt (104 mg, 0.2 mmol) was dissolved in acetonitrile (16 ml) and potassium carbonate (276 mg, 2 mmol) was added. 4-chloro-cinnamyl chloride (45 mg, 0.22 mmol) was dissolved in acetonitrile (4 ml) and added to the previous solution. The reaction mixture was stirred at 70°C for 4 hours then cooled to room temperature. Diethyl ether (50 ml) was added, the organic layer was washed with water then concentrated *in vacuo*. The residue was purified by column chromatography (hexane:ethyl acetate 50:50 +1% Et3N) to afford the title product (87 mg, 85%). MS (ES+) 512/514/516 M+H. 1H NMR (500 MHz, CDCl­3) 1.70 (m, 2H), 1.95 (m, 4H), 3.00 (m, 2H), 3.20 (m, 2H), 3.80 (br s, 2H), 6.25 (m, 1H), 6.50 (d, 1H), 7.20-7.40 (m, 7H), 7.48 (s, 1H), 8.15 (br s, 1H), 8.60 (d, 1H). 13C NMR (125 MHz, CDCl­3) 36.5, 43.5, 50.6, 60.4, 61.1, 118.9, 119.5, 121.8, 123.1, 127.0, 127.5, 128.4, 128.7, 132.0, 133.2, 135.2, 150.7, 152.5, 164.3.

**General synthetic method (path b): synthesis of 1-(2-Chloropyridin-4-yl)carbonyl-1’-[*trans*-3-(4-chlorophenyl)allyl]-5-fluorospiro[indoline-3,4’-piperidine] 3**

##### Step 1: Preparation of 8-[*trans*-3-(4-chlorophenyl)allyl]-1,4-dioxa-8-azaspiro[4.5]decane

1,4-Dioxa-8-azaspiro[4.5]decane (0.88 g, 6.15 mmol) was dissolved in chloroform (5 ml) and diisopropylethylamine (2.1 ml, 12 mmol) was added. A solution of 4-chlorocinnamyl chloride (1.2 g, 6.4 mmol) dissolved in chloroform (2 ml) was added and mixture was heated to 70 °C overnight. The solvents were evaporated *in vacuo* and flash chromatography [SiO2; ethyl acetate-hexane-triethylamine (50:50:2)] afforded the title product as a yellow oil (1.38 g, 76%). 1H NMR (400 MHz, CDCl­3) 1.78 (t, J = 4 Hz, 4H), 2.60 (br s, 4H), 3.18 (d, J = 5 Hz, 2H), 3.96 (s, 4H), 6.27 (dt, J = 12 & 5 Hz, 1H) 6.47 (d, J = 12 Hz, 2H), 7.28, m, 4H). MS (ES+) 294/296 M+H+.

##### Step 2: Preparation of 1-[*trans*-3-(4-chlorophenyl)allyl]-4-oxopiperidine

8-[*trans*-3-(4-Chlorophenyl)allyl]-1,4-dioxa-8-azaspiro[4.5]decane (1.38 g, 4.7 mmol) was dissolved in methanol (20 ml) and 6 N hydrochloric acid (80 ml) was added. The mixture was heated to reflux for 4 h. The mixture was cooled and brought to pH 14 with solid sodium hydroxide pellets (CARE! Exothermic), the solution becoming opaque. The aqueous was extracted three times with ether. The organics were washed with brine, dried over anhydrous MgSO4 and evaporated to give the desired ketone (1.17g , 100%) 1H NMR (400 MHz, CDCl­3) 2.38 (m, 4H), 2.70 (m, 4H), 3.15 (d, J = 5 Hz, 2H), 3.96 (s, 4H), 6.17 (dt, J = 12 & 5 Hz, 1H), 6.40 (d, J = 12 Hz, 1H), 7.20 (m, 4H). MS (ES+) 250/252 M+H+.

##### Step 3: Preparation of 1-[*trans*-3-(4-chlorophenyl)allyl]-4-methoxymethylenepiperidine

Methoxymethyltriphenylphosphonium chloride (2.4 g, 7 mmol) was dissolved in tetrahydrofuran (20 ml) and was cooled to 4 ºC. Potassium tert-butoxide (0.78 g, 7 mmol) was added, turning the solution a bright orange colour. The reaction was left as such for 30 min. A solution of 1-[*trans*-3-(4-chlorophenyl)allyl]-4-oxopiperidine (0.85 g, 7 mmol) dissolved in tetrahydrofuran (10 ml) was added and the mixture was stirred for 10 min. The solvents were evaporated *in vacuo* and the residue re-suspended in ether. The organics were washed with water and dried over anhydrous magnesium sulfate. Flash chromatography [SiO2; ethyl acetate-hexane-triethylamine (50:50:2)] gave the desired enol ether (0.85 g, 89%). 1H NMR (400 MHz, CDCl­3) 2.10 (t, J = 6 Hz, 2H), 2.35 (t, J = 6 Hz, 2H), 2.4 (m, 4H), 3.13 (d, J = 5 Hz, 2H), 3.55 (s, 3H), 5.80 (s, 1H), 6.30 (dt, J = 11 & 5 Hz, 1H), 6.45 (d, J = 11 Hz, 1H), 7.28 (m, 4H). MS (ES+) 278/280 (M+H+).

Step 4: 1’-[*trans*-3-(4-chlorophenyl)allyl]-5-fluorospiro[indoline-3,4’-piperidine]

To a solution of 1-[*trans*-3-(4-chlorophenyl)allyl]-4-methoxymethylenepiperidine (20g, 72 mmol) and 4-fluoro-phenylhydrazine (11.7 g, 72 mmol) in chloroform (720 ml) at room temperature was added trifluoroacetic acid dropwise (36 ml). The reaction mixture was stirred at 75°C for 3 hours ; the reaction mixture was cooled to 45°C and trifluoroacetic acid was added again dropwise (36 ml). The solution was stirred at 90°C for 3h30 then colled to 35°C. Triethylsilane (10 ml, 320 mmol) was added dropwise and the reaction mixture was stirred at 55°C for 12 hours. The reaction mixture was cooled to room temperature, then poured into concentrated ammonia solution (80 ml) in ice water (50 ml). The organic layer was separated and the aqueous layer was extracted four times with dichloromethane. Combined organic layers were dried over sodium sulphate and concentrated *in vacuo*. The residue was purified by column chromatography (hexane:ethyl acetate 70:30 +1% Et3N) to afford the title product (23 g, 92%). 1H NMR (300 MHz, CDCl­3) 1.55 (d, J = 12 Hz, 2H), 1.70 (dt, J = 3 and 12 Hz, 2H), 1.90 (t, J = 12 Hz, 2H), 2.72 (d, J = 12 Hz, 2H), 2.97 (d, J = 9 Hz, 2H), 6.06 (td, J = 8 and 15 Hz, 1H), 6.27 (d, J = 15 Hz, 1H), 6.32 (m, 1H), 6.50 (m, 1H), 6.55 (m, 1H), 7.10 (d, J = 5 Hz, 1H); MS (ES+) 357/359 (M+H+).

Step 5: 1-(2-Chloropyridin-4-yl)carbonyl-1’-[*trans*-3-(4-chlorophenyl)allyl]-5-fluorospiro[indoline-3,4’-piperidine]

To a solution of 1’-[*trans*-3-(4-chlorophenyl)allyl]-5-fluorospiro[indoline-3,4’-piperidine] (230 mg, 0.75 mmol) and triethylamine (0.2 ml) in dichloromethane (5 ml) at 0°C was added 2-chloroisonicotinoyl chloride (0.12 g, 0.7 mmol) in dichloromethane (5 ml). The reaction mixture was stirred at room temperature for 2 hours then poured into 1M aqueous sodium bicarbonate (8ml). The organic layer was separated, the aqueous layer was extracted twice with chloroform (8ml), the combined organic layers were dried over magnesium sulfate then concentrated *in vacuo*. The residue was purified by column chromatography (hexane:ethyl acetate 50:50 +1% Et3N) to afford the title product (0.26 g, 81%). MS (ES+) 496/498 (M+H+); 1H NMR (500 MHz, CDCl­3) 1.70 (m, 2H), 1.95 (m, 4H), 3.00 (m, 2H), 3.15 (m, 2H), 3.80 (br s, 2H), 6.25 (m, 1H), 6.50 (d, 1H), 6.95 (dd, 1H), 7.30-7.40 (m, 7H), 7.48 (s, 1H), 8.20 (br s, 1H), 8.60 (d, 1H). 13C NMR (125 MHz, CDCl­3) 36.4, 48.5, 50.6, 60.5, 61.1, 109.9, 114.8, 118.9, 119.5, 121.8, 126.9, 127.5, 128.7, 132.1, 133.2, 135.2, 137.0, 146.9, 150.7, 152.5, 164.4.

**General synthetic method (path c): synthesis of 5-chlorospiro[indoline-3,4’-(1’,2’,3’,4’-tetrahydropyridine]-1’-carboxylic acid *tert*-butyl ester**

Step 1: 4-{[(4-Chloro-2-iodo-phenyl)-(2,2,2-trifluoro-acetyl)-amino]-methyl}-3,6-dihydro-2H-pyridine-1-carboxylic acid ester

tert-butyTriphenylphosphine (5.16 g, 19.7 mmol) was dissolved in tetrahydrofuran (130 ml) and the solution was cooled to 0°C under argon. Diisopropylazodicarboxylate (3.82 ml, 19.7 mmol) was added dropwise over 10 min and the resulting mixture was stirred at 0°C for 20 min (formation of a white precipitate). N-(4-Chloro-2-iodo-phenyl)-2,2,2-trifluoro-acetamide (5.5 g, 15.7 mmol) was added as a solid, followed by 4-hydroxymethyl-3,6-dihydro-2H-pyridine-l -carboxylic acid tert-butyl ester [47] (3.4 g, 15.7 mmol) dissolved in a minimum volume of tetrahydrofuran. The reaction mixture was allowed to warm to room temperature and stirred for 12 hours. The solution was then concentrated *in vacuo* and the residue subjected to silica gel chromatography (cyclohexane:ethyl acetate 9: 1) to afford 4-{[(4- chloro-2-iodo-phenyl)-(2,2,2-trifluoro-acetyl)-amino]-methyl}-3,6-dihydro-2H-pyridine-l- carboxylic acid tert-butyl ester (4.7 g). 1H NMR (300 MHz, CDC13) 1.5 (s, 9H), 2.20 (m, 2H), 3.49 (m, 1H), 3.50 (d, J = 17 Hz, 1H), 3.55 (m, 1H), 3.8-3.9 (m, 2H), 5.02 (d, j = 17 Hz, 1H), 5.40 (s, 1H), 7.0 (m, 1H), 7.38 (dd, 1H), 7.92 (d, 1H); MS (ES+) 445/447 (M+H+-CO2- isobutene), 486/488 (M+H+-isobutene).

Step 2: 5-chlorospiro[indoline-3,4’-(1’,2’,3’,4’-tetrahydropyridine]-1’-carboxylic acid *tert*-butyl ester

In a dried, argon purged flask, 4- {[(4-chloro-2-iodo-phenyl)-(2,2,2-trifluoro- acetyl)-amino] methyl }-3,6-dihydro-2H-pyridine-1-carboxylic acid tert-butyl ester obtained in Step 1 (3.55 g, 6.52 mmol) was dissolved in dimethylformamide (55 ml); triethylamine (2.3 ml, 16.3 mmol), tetrabutylammonium bromide (2.5 g, 7.8 mmol) and palladium(II) acetate (0.22 g, 0.97 mmol) were successively added and the solution was heated at 80°C for 3 hours. After cooling to room temperature, the reaction mixture was diluted with ethyl acetate, washed with brine, dried over sodium sulphate and concentrated *in vacuo*. The crude residue was dissolved in methanol (140 ml) and water (30 ml), placed under argon and potassium carbonate (6.8 g) was added. The reaction mixture was stirred for 1 hour at room temperature, the mixture was filtered and the filtrate concentrated in vacuo. The residue was diluted with ethyl acetate, washed with brine, dried (sodium sulphate) and concentrated in vacuo. Silica gel chromatography of the residue (cyclohexane:ethyl acetate 8:2) afforded 5-chloro-spiro[indoline-3,4'-(1’,2',3',4'- tetrahydropyridine)]-1 ' carboxylic acid tert-butyl ester (1.3 g, 62%) as a a pale yellow powder. M.p. 50-51°C; 1H NMR (600 MHz, CDCl2CDCl2, 80°C) 1.54 (s, 9H), 1.83 (m, 1H), 1.99 (m, 1H), 3.36 (d, J = 1 1.4 Hz, 1H), 3.50 (d, J = 11.4 Hz, 1H), 3.51 (m, 1H), 3.75 (brs, 1H, NH), 3.78 (, IH), 4.81 (d, J = 8.6 Hz, 1H), 6.57 (d, J - 10.2 Hz, 1H), 6.98 (d, J = 2.4 Hz, 1H), 7.02 (m, 1H), 7.03 (dd, J = 10.2, 2.4 Hz, 1H); 13C NMR (125 MHz, CDCl2CDCl2, 80°C) selected data 28.2, 33.2, 39.1, 60.6, 108.5, 110.2, 123.5, 126.4, 127.5; MS (ES+) 221/223 (M+H+-CO2-isobutene), 265/267 (M+H+-isobutene); 321/323 (M+H+).

**Synthesis of the radioligand 1-(2-chloropyridin-4-yl)carbonyl-1’-[*trans*-3-(4-chlorophenyl)allyl]-5-chlorospiro[indoline-3,4’-piperidine] [3H]-2**

Step 1: 2’,3’-[3H]-5-chlorospiro[indoline-3,4’-piperidine]-1’-carboxylic acid *tert*-butyl ester

A solution of 5-chlorospiro[indoline-3,4’-(1’,2’,3’,4’-tetrahydropyridine]-1’-carboxylic acid *tert*-butyl ester (26.6 mg, 82.9 µmol) and 5% Rh/C (29.7 mg) in methanol (3 ml) was treated with tritium gas (1.7 bar) for 2 hours. The reaction mixture was purged with nitrogen, lyophilized, diluted three times with methanol (1 ml) and lyophilized again. The residue was dissolved in ethanol and the catalyst removed by filtration. The solvent was evaporated *in vacuo* and the product was purified by column chromatography (hexane/ethyl acetate 2:1) to afford the title product in 95% radiochemical purity at a specific activity of 1.64 TBq.mmol-1.

Step 2: 2’,3’-[3H]- 1-(2-chloropyridin-4-yl)carbonyl-1’-[*trans*-3-(4-chlorophenyl)allyl]-5-chlorospiro[indoline-3,4’-piperidine]

To a solution of 2’,3’-[3H]-5-chlorospiro[indoline-3,4’-piperidine]-1’-carboxylic acid *tert*-butyl ester prepared in step 1 (230 mCi) and triethylamine (60 µl) in chloroform (2 ml) at 0°C was added dropwise 2-chloroisonicotinoyl chloride (prepared from 2-chloroisonicotinic acid (75 mg) and thionyl chloride (330 µl) in 2ml hexane) in chloroform (1 ml). The reaction mixture was stirred at room temperature for 2 hours then poured into 1M aqueous sodium bicarbonate (2 ml). The organic layer was separated, the aqueous layer was extracted with chloroform (10 ml), the combined organic layers were dried over magnesium sulfate then concentrated *in vacuo*. The residue was dissolved in dichloromethane (5 ml) and trifluoroacetic acid (250 µl) was added. The solution was stirred at room temperature for 12 hours then concentrated *in vacuo*. The crude residue was dissolved in acetonitrile (5 ml) then potassium carbonate (185 mg) was added followed by a solution of 4-chloro-cinnamyl chloride (30 mg) in acetonitrile (2 ml). The solution was stirred at 70°C for 4 hours then cooled to room temperature. Water (2 ml) was added then the mixture extracted with diethyl ether (15 ml). The organic layer was dried over magnesium sulphate and concentrated *in vacuo*. The residue was purified by preparative HPLC (SymmetryShield RP8, water/acetonitrile 1:1, 1 ml.min-1) to afford two fractions of the title product showing total activity of 75mCi and 54 mCi respectively.

Mass spectra were recorded on a ZMD (Micromass, Manchester UK) or a ZQ (Waters Corp. Milford, MA, USA) mass spectrometer equipped with an electrospray source (ESI; source temperature 80 to 100°C; desolvation temperature 200 to 250°C; cone voltage 30 V; cone gas flow 50 l.hr-1, desolvation gas flow 400 to 600 l.hr-1, mass range: 150 to 1000 Da).

Physical chemistry measurements.

Basic pKa was measured by UV-spectrophotometric titration, using Sirius GL-pKa with DPAS UV/vis probe, at 20°C. Because the low solubility of many compounds does not allow titration in 100% aqueous solution, titrations are carried out in mixed methanol-aqueous solutions (typically with 20-50% methanol) and the values obtained are used to calculate 100% aqueous pKa values using Yasuda-Shedlovsky extrapolation.

logP was measured by retention time on reversed-phase HPLC using octanol coated mini-columns. Retention times are compared with a series of standards of known logP. The upper limit of logP measurement by this technique is normally about 5.0, but many spiroindoline compounds are higher than this in the uncharged form. By buffering the aqueous eluent at pH values below the pKa (typically at pH5-6) distribution coefficients (logD) are measured which are then corrected for the degree of ionisation using previously measured basic pKa values.

For solubility measurement, saturated solutions were prepared by 24-hour roller shaking of buffered aqueous samples (typically 1mg.ml-1), followed by one hour equilibribration at lab temperature (20°C). Liquid samples were then phase-separated by centrifugation and solid samples by filtration though 0.47 microm Millipore HV4 filters. Samples are then compared by HPLC with standard samples of known concentration.

Photostability was measured by irradiation of thin film deposits on glass in an Atlas Suntest xenon lamp sytem. Compounds are typically applied at 2 mg.cm-2 from solutions of technical material dissolved in acetone or acetonitrile, or of formulated material in aqueous dilution. After exposure, remaining compound, and any photolysis products, is washed from the slides and analyzed by HPLC to generate loss v exposure time curves. These are examined by eye to generate T50 values (the time for loss of the first 50% of the active ingredient).

### References specific to Supplementary Text.

41 Keen M (1997) Radioligand-Binding Methods for Membrane Preparations and Intact Cells. Methods Mol Biol 83: 1-24.

42 Motulsky, H. J. (1999) Analyzing Data with GraphPad Prism (GraphPad Software Inc., San Diego CA). http://graphpad.com/manuals/analyzingdata.pdf

43 Alfonso A, Grundahl K, McManus JR, Asbury JM, Rand JB (1994) Alternative Splicing Leads to Two Cholinergic Proteins in Caenorhabditis elegans. J Mol Biol 241: 627-630.

44 Kitamoto T, Wang W, Salvaterra PM (1998) Structure and Organization of the Drosophila Cholinergic Locus. J Biol Chem 273: 2706-2713.

45 Spradling AC, Rubin GM (1982) Transposition of cloned P elements into Drosophila germ line chromosomes. Science 218: 341-347.

46 Ashton WD (1972) The logit transformation: with special reference to its uses in bioassay. London: Lubrecht & Cramer Ltd.

47 Hughes, R. C., Dvorak, C. A. & Meyers, A. I. (2001) An Asymmetric Approach to Spirocylic Systems: A Formal Synthesis of Zizaene.J Org Chem 66: 5545-5551.
