# Supplementary material for: Spiroindolines Identify the Vesicular Acetylcholine Transporter as a Novel Target for Insecticide Action
Source: PLoS One. 2012 May 1;7(5):e34712. doi: 10.1371/journal.pone.0034712 (PMC3341389; doi:10.1371/journal.pone.0034712)
Supplement: Table S1 — Insecticidal activity of a selected set of spiroindoline compounds against lepidopteran larvae. Activity is given as the concentration giving 80% mortality (EC80 (µg.ml-1)).Use of < and ≤ indicate that the operator judges the true EC80 to be well below and a little below, respectively, the given concentration but above the next lowest concentration tested. (DOC) [file pone.0034712.s002.doc]

**Table S1. Insecticidal activity of a selected set of spiroindoline compounds against lepidopteran larvae.**

|  |  |
| --- | --- |

Activity is given as the concentration giving 80% mortality (EC_80_ (µg.ml^-1^)).Use of < and ≤ indicate that the operator judges the true EC80 to be well below and a little below, respectively, the given concentration but above the next lowest concentration tested.
