# Supplementary material for: Spiroindolines Identify the Vesicular Acetylcholine Transporter as a Novel Target for Insecticide Action
Source: PLoS One. 2012 May 1;7(5):e34712. doi: 10.1371/journal.pone.0034712 (PMC3341389; doi:10.1371/journal.pone.0034712)
Supplement: Table S2 — Structures and biological activities of spiroindoline analogues. Activity is given as the concentration giving 80% mortality (EC80 (µg.ml-1)).Use of < and ≤ indicate that the operator judges the true EC80 to be well below and a little below, respectively, the given concentration but above the next lowest concentration tested. (DOC) [file pone.0034712.s003.doc]

Table S2. Structures and biological activities of spiroindoline analogues


| **entry** | **R_1_** | **R_2_** | **R_3_** | *Spodoptera littoralis L1* | *Heliothis virescens L1* | *Plutella xylostella L2* |
| --- | --- | --- | --- | --- | --- | --- |
| **1** | H | 5-Cl | H | >200 | >200 | >200 |
| **2** | H | 5,7-diCl |  | >200 | =50 | >200 |
| **3** | H | 5-Cl |  | >200 | >200 | >200 |
| **4** | H | 7-Br |  | =200 | <200 | >200 |
| **5** | H | 7-Cl |  | =200 | =200 | >200 |
| **6** | H | 7-Me |  | =200 | =200 | >200 |
| **7** | Ac |  |  | >200 | >200 | >200 |
| **8** | Ac |  |  | >200 | >200 | >200 |
| **9** | Ac | 5-Cl |  | =50 | =12 | <200 |
| **10** | Ac | 5-F |  | =50 | =12 | <50 |
| **11** | Ac |  |  | >200 | >200 | >200 |
| **12** | CO_2_Me | 5-F |  | >12 | =12 | =200 |
| **13** | CONHMe | 5-F |  | =12 | =12 | =50 |
| **14** | COCH_2_CF_3_ | 5-F |  | <50 | =50 | =50 |
| **15** | SO_2_CH_2_CH_3_ | 5-F |  | >200 | =50 | >200 |
| **16** |  | 5-F |  | =50 | =50 | <50 |
| **17** |  | 5-F |  | <200 | =50 | =12/=200 |
| **18** |  | 5-Cl | H | >200 | >200 | >200 |
| **19** |  | 5-Cl | Me | >200 | >200 | >200 |
| **20** |  | 5-Cl |  | >200 | >200 | >200 |
| **21** |  | 5-Cl |  | >200 | >200 | >200 |
| **22** |  | 5-Cl |  | =200 | =200 | >200 |
| **23** |  | 5-F |  | <200 | <50 | =50 |
| **24** |  | H |  | =200 | =50 | =200 |
| **25** |  |  |  | >200 | >12 | >200 |
| **26** (SYN351) |  | 5-Cl |  | =12 | =0.8 | <12 |
| **27**  (SYN876) |  | 5-F |  | =12 | =3 | =3 |
| **28** |  | 5,7-diF |  | =200 | =3 | =12 |
| **29** |  | 6-F |  | =12 | =12 | =3 |
| **30** |  | 5-Cl |  | <200 | =12 | >200 |
| **31** |  | 5-F |  | <50 | =12 | <50 |
| **32** |  | 5-Cl |  | <50 | =12 | =12 |
| **33** |  | 5-Cl |  | <50 | =3 | =12 |
| **34** |  | 5-Cl |  | =12 | =0.8 | <3 |
| **35** |  | 5-F |  | =50 | =3 | <12 |
| **36** |  | 5-F |  | =50 | =3 | =12 |
| **37** |  |  |  |  | <12 | =50 |
| **38** |  | 5-F |  | =200 | =50 | >200 |
| **39** |  | 5-Cl |  | =200 | >200 | >200 |
| **40** |  | 5-Cl |  | =50 | =3 | <50 |
| **41** |  | 5-Cl |  | =50 | =12 | =12 |
| **42** |  | 5-Cl |  | =50 | <50 | =200 |
| **43** |  | 5-Cl |  | =50 | =12 | =200 |
| **44** |  | 5-Cl |  | =50 | =3 | =50 |
| **45** |  | 5-Cl |  | >200 | =200 | =200 |
| **46** |  | 5-Cl |  | >200 | =50 | =200 |
| **47** |  | 5-F |  | >200 | >200 | >200 |
| **48** |  | 5-F |  | >200 | =200 | >200 |
| **49** |  | 5-F |  | >200 | =200 | >200 |
| **50** |  | 5-F |  | >200 | =200 | >200 |
| **51** |  | 5-Cl |  | >200 | >200 | >200 |
| **52** |  | 5-Cl |  | >200 | >200 | >200 |
| **53** |  | 5-Cl |  | >200 | >200 | >200 |
| **54** |  | 5-Cl |  | <200 | <50 | <50 |
| **55** |  | 5-Cl |  | >200 | >200 | >200 |
| **56** |  | 5-Cl |  | >200 | >200 | >200 |
| **57** |  | 5-Cl |  | >200 | >200 | >200 |
| **58** |  | 5-F |  | >200 | >200 | >200 |
| **59** |  | 5-F |  | >200 | >200 | >200 |
| **60** |  | 5-F |  | >200 | >200 | >200 |
| **61** |  | 5-F |  | >200 | >200 | >200 |
| **62** |  | 5-F |  | >200 | >200 | >200 |
| **63** |  | 5-F |  | =500 | >500 | >500 |
| **64** |  | 5-F |  | =12 | <3 | <12 |
| **65** |  | 6-OCF_3_ |  | =200 | <12 | <12 |
| **66** |  |  |  | >200 | =50 | >200 |
| **67** |  | 5-F |  | >200 | >200 | >200 |
| **68** |  | 5-F |  | >200 | >200 | >200 |
| **69** |  | 5-F |  | >200 | =200 | =200 |
| **70** |  | 5-F |  | =12 | =3 | =12 |
| **71** |  | 5-Cl |  | =200 | =50 | >200 |
| **72** |  | 5-F |  | =50 | =12 | =50 |
| **73** |  | 5-F |  | =50 | =12 | =50 |
| **74** |  | 5-Cl |  | <12 | <3 | <12 |
| **75** |  | 5-F |  | >200 | >200 | >200 |
| **76** |  | 5-F |  | <200 | =50 | <50 |
| **77** |  | 5,7-diCl |  | <200 | =12 | =12 |
| **78** |  | 5-F |  | >200 | =200 | >200 |

b)

| **entry** | **Core structure** | **R_1_** | **R_2_** | *Spodoptera littoralis L1* | *Heliothis virescens L1* | *Plutella xylostella L2* |
| --- | --- | --- | --- | --- | --- | --- |
| **79** |  |  |  | =12 | =3 | =12 |
| **80** |  |  |  | =50 | =12 | <200 |
| **81** |  |  |  | >200 | =12 | >200 |
| **82** |  |  |  | <12 | <12 | =50 |
| **83** |  |  |  | >200 | >200 | >200 |
| **84** |  |  |  | =200 | =12 | =50 |
| **85** |  | CH_3_ |  | =200 | <50 | >200 |
| **86** |  |  |  | =12 | =12 | =200 |
| **87** |  |  |  | =200 | <50 | =50 |
| **88** |  |  |  | >200 | >200 | >200 |
| **89** |  |  |  | >200 | =200 | >200 |
| **90** |  |  |  | =200 | =50 | >200 |
| **91** |  |  |  | >200 | =200 | >200 |
| **92** |  |  |  | >200 | =12 | >200 |
| **93** |  |  |  | =50 | <12 | =200 |
| **94** |  | - |  | >200 | =50 | >200 |
| **95** |  | - |  | >200 | <50 | =200 |
