# Supplementary material for: Spiroindolines Identify the Vesicular Acetylcholine Transporter as a Novel Target for Insecticide Action
Source: PLoS One. 2012 May 1;7(5):e34712. doi: 10.1371/journal.pone.0034712 (PMC3341389; doi:10.1371/journal.pone.0034712)
Supplement: Table S3 — Potency of spiroindoline analogues for displacement of Spiroindoline binding and inhibition of vesicular acetylcholine uptake. Columns headed with the insect species names show data for displacement of [3H]-SYN876 binding. Acetylcholine uptake was measured using a fraction isolated from PC12 cells expressing Drosophila VAChT. Displacement and inhibition assays are described in the Text S1. Missing values were not determined. Some values are ranges or approximations (∼) based on a limited number of concentrations tested; others were determined by curve fitting as described in Text S1. Compound numbers refer to the structures in Table S2. (DOC) [file pone.0034712.s004.doc]

Table S3. Potency of spiroindoline analogues for displacement of Spiroindoline binding and inhibition of vesicular acetylcholine uptake.

| **Compound** | ***L. sericata* IC50 nM** | ***L.migratoria* IC50 nM** | | ***S. littoralis* IC50 nM** | | **Acetylcholine uptake  IC50 nM** |
| --- | --- | --- | --- | --- | --- | --- |
| **1** | inactive | 2500 | 2778 | | inactive | |
| **2** | 9.7 | 24 |  | |  | |
| **3** | 22.0 | 19 | 31 | | 695.0 | |
| **4** | 0.4 | 3 | 1 - 10 | |  | |
| **5** | 1.8 | 2 | <10 | |  | |
| **6** | 1.7 | 7 |  | |  | |
| **7** | 453.5 |  |  | |  | |
| **8** | 553.0 |  |  | |  | |
| **9** | 0.7 | 0.9 | 0.03 | | 36.1 | |
| **10** | 0.5 |  |  | |  | |
| **11** | 386.6 |  |  | |  | |
| **12** | 0.7 | 0.4 | 0.1 | | 4.2 | |
| **13** | 0.8 |  |  | |  | |
| **14** | 1.5 | 0.4 | 4.3 | | 8.7 | |
| **15** | 1.6 |  | ~1 | |  | |
| **16** | 1.7 |  |  | |  | |
| **17** | 0.9 | 0.9 | 0.14 | | 3.3 | |
| **18** | 239.0 | 100 | 52 | | 240.0 | |
| **19** | 100.0 |  |  | |  | |
| **20** | 0.5 |  | <10 | |  | |
| **21** | 0.7 |  |  | |  | |
| **22** | 1.0 |  | 1 - 10 | |  | |
| **23** | 0.6 |  |  | |  | |
| **24** | 0.5 | 0.5 |  | |  | |
| **25** | 1.0 |  |  | |  | |
| **26** | 2.0 | 0.7 | 0.2 | | 0.8 | |
| **27** | 1.4 | 0.7 | 0.33 | | 4.8 | |
| **28** | 3.6 |  |  | | 5.1 | |
| **29** | 2.5 |  |  | | 1.8 | |
| **30** | 1.1 |  |  | |  | |
| **31** | 0.6 |  |  | | 1.3 | |
| **32** | 1.9 |  |  | | 3.4 | |
| **33** | 0.7 |  |  | |  | |
| **34** | 1.9 |  |  | |  | |
| **35** | 1.5 |  |  | |  | |
| **36** | 1.1 |  |  | |  | |
| **37** | 28.5 |  |  | |  | |
| **38** | 0.8 |  | <10 | |  | |
| **39** | 0.6 |  |  | | <1uM | |
| **40** | 0.6 |  |  | |  | |
| **41** | 4.8 |  |  | |  | |
| **42** | 1.6 |  |  | |  | |
| **43** | 9.6 |  |  | |  | |
| **44** | 0.7 |  |  | |  | |
| **45** | 208.5 |  |  | |  | |
| **46** | 11.0 |  |  | |  | |
| **47** | 2.3 |  |  | |  | |
| **48** | 71.8 |  |  | |  | |
| **49** | 0.6 |  |  | |  | |
| **50** | 27.0 |  |  | |  | |
| **51** | 57.0 | 12, 85 | 4.5 | | 68.7 | |
| **52** | 6.4 | 9 | 0.7 | | 8.8 | |
| **53** | 247.5 | 93 | 63 | | 88.0 | |
| **54** | 0.6 |  |  | |  | |
| **55** | 30.2 |  |  | |  | |
| **56** | 367.0 |  |  | |  | |
| **57** | 92.9 |  |  | |  | |
| **58** | 1.6 |  | <10 | |  | |
| **59** | 1.7 |  | <10 | |  | |
| **60** | 2.3 |  |  | |  | |
| **61** | 7.8 |  |  | |  | |
| **62** | 90.5 |  |  | |  | |
| **63** | 40.1 |  |  | |  | |
| **64** | 0.9 |  |  | |  | |
| **65** | 1.5 |  |  | |  | |
| **66** | 6.0 |  |  | |  | |
| **67** | 1.0 |  |  | |  | |
| **68** | 2000.0 |  |  | |  | |
| **69** | 2.4 |  |  | |  | |
| **70** | 8.8 | 4 |  | |  | |
| **71** | 5.5 | 10 |  | |  | |
| **72** | 2.1 |  |  | | 0.4 | |
| **73** | 4.7 |  |  | |  | |
| **74** | 1.9 | 1.7 |  | |  | |
| **75** | 8.7 | 178 |  | |  | |
| **76** | 0.5 | 1 |  | |  | |
| **77** | 13.1 |  |  | |  | |
| **78** | 26.4 |  |  | |  | |
| **79** | 2.3 | 2.9 |  | |  | |
| **80** | 85.3 |  |  | |  | |
| **81** | 7.2 |  |  | |  | |
| **82** | 40.7 |  |  | |  | |
| **83** | 816.6 | 962 |  | |  | |
| **84** | 3.9 |  |  | |  | |
| **85** | 1.0 | 3 |  | |  | |
| **86** | 1.9 |  |  | | 0.8 | |
| **87** | 7.6 |  |  | |  | |
| **88** | 6.8 |  |  | |  | |
| **89** | 15.2 |  |  | |  | |
| **90** | 1.2 |  |  | |  | |
| **91** | 6.8 |  |  | |  | |
| **92** | 17.4 |  |  | |  | |
| **93** | 2.9 |  |  | |  | |
| **94** | 0.5 |  | 0.1 - 1 | |  | |
| **95** | 0.6 |  |  | |  | |

Columns headed with the insect species names show data for displacement of [^3^H]-SYN876 binding. Acetylcholine uptake was measured using a fraction isolated from PC12 cells expressing Drosophila VAChT. Displacement and inhibition assays are described in the Text S1. Missing values were not determined. Some values are ranges or approximations (~) based on a limited number of concentrations tested; others were determined by curve fitting as described in Text S1. Compound numbers refer to the structures in Table S2.
