# Supplementary material for: Spiroindolines Identify the Vesicular Acetylcholine Transporter as a Novel Target for Insecticide Action
Source: PLoS One. 2012 May 1;7(5):e34712. doi: 10.1371/journal.pone.0034712 (PMC3341389; doi:10.1371/journal.pone.0034712)
Supplement: Table S4 — Pharmacological selectivity of the Spiroindoline binding site in L. Sericata. Compounds were assayed for [3H]-SYN876 displacement as described in Text S1. Compounds were scored as inactive if they gave less that 20% displacement at the highest test concentration (nominally 10 µM). (DOC) [file pone.0034712.s005.doc]

Table S4. Pharmacological selectivity of the Spiroindoline binding site in *L. sericata*

| **Test chemical** | **Displacement of [^3^H]-SYN876** |
| --- | --- |
| aldicarb | Inactive |
| profenofos | Inactive |
| pirimiphos-methyl | Inactive |
| thiamethoxam | Inactive |
| nitenpyram | Inactive |
| imidacloprid | Inactive |
| N-methyllycaconitine | Inactive |
| spinosyn A | Inactive |
| atropine | Inactive |
| dieldrin | Inactive |
| endrin | Inactive |
| fipronil | Inactive |
| emamectin | 32% @ 10µM |
| abamectin | Inactive |
| cypermethrin | Inactive |
| lambdacyhalothrin | Inactive |
| fenvalerate | Inactive |
| N-decarboxymethylated indoxacarb | Inactive |
| metaflumizone | Inactive |
| ryanodine | Inactive |
| flubendiamide | Inactive |
| chlorantraniliprole | Inactive |
| chlorfenapyr | Inactive |
| diafenthiuron | Inactive |
| antimycin A1 | Inactive |
| fenoxycarb | Inactive |
| pyriproxyfen | Inactive |
| chlordimeform | Inactive |
| amitraz | Inactive |
| pyridalyl | Inactive |
| spirotetramat | Inactive |
| lufenuron | Inactive |
| chlorfluazuron | Inactive |
| buprofezin | Inactive |
| pymetrozine | Inactive |
| halofenazide | Inactive |
| SYN876 | IC50 = 0.001 µM |
| pinacidil | Inactive |
| methotrexate | Inactive |
| domoic acid | Inactive |
| veratrine | Inactive |
| (±)-Vesamicol | IC50 = 1.2 µM |
| (±)-4-Amino benzovesamicol | IC50 = 0.009 µM |

Compounds were assayed for [3H]-SYN876 displacement as described in Text S1. Compounds were scored as inactive if they gave less that 20% displacement at the highest test concentration (nominally 10 µM).
